# Synthesis of Stable NAD^+^ Mimics as Inhibitors for the *Legionella pneumophila* Phosphoribosyl Ubiquitylating Enzyme SdeC

**DOI:** 10.1002/cbic.202000230

**Published:** 2020-06-16

**Authors:** Jerre M. Madern, Robbert Q. Kim, Mohit Misra, Ivan Dikic, Yong Zhang, Huib Ovaa, Jeroen D. C. Codée, Dmitri V. Filippov, Gerbrand J. van der Heden van Noort

**Affiliations:** ^1^ Leiden Institute of Chemistry Leiden University Einsteinweg 55 2333 CC Leiden The Netherlands; ^2^ Oncode Institute and Department of Cell and Chemical Biology Leiden University Medical Centre Einthovenweg 20 2333 ZC Leiden The Netherlands; ^3^ Institute of Biochemistry II Goethe University Faculty of Medicine Theodor-Stern-Kai 7 60590 Frankfurt am Main Germany; ^4^ Department of Pharmacology and Pharmaceutical Sciences School of Pharmacy University of Southern California 1985 Zonal Avenue Los Angeles CA 90089 USA; ^5^ Buchmann Institute for Molecular Life Sciences Goethe University Frankfurt, Riedberg Campus Max-von-Laue-Strasse 15 60438 Frankfurt am Main Germany

**Keywords:** ADP-ribosylation, inhibitors, Legionella, ubiquitylation

## Abstract

Stable NAD^+^ analogues carrying single atom substitutions in either the furanose ring or the nicotinamide part have proven their value as inhibitors for NAD^+^‐consuming enzymes. To investigate the potential of such compounds to inhibit the adenosine diphosphate ribosyl (ADPr) transferase activity of the Legionella SdeC enzyme, we prepared three NAD^+^ analogues, namely carbanicotinamide adenosine dinucleotide (c‐NAD^+^), thionicotinamide adenosine dinucleotide (S‐NAD^+^) and benzamide adenosine dinucleotide (BAD). We optimized the chemical synthesis of thionicotinamide riboside and for the first time used an enzymatic approach to convert all three ribosides into the corresponding NAD^+^ mimics. We thus expanded the known scope of substrates for the NRK1/NMNAT1 enzyme combination by turning all three modified ribosides into NAD^+^ analogues in a scalable manner. We then compared the three NAD^+^ mimics side‐by‐side in a single assay for enzyme inhibition on Legionella effector enzyme SdeC. The class of SidE enzymes to which SdeC belongs was recently identified to be important in bacterial virulence, and we found SdeC to be inhibited by S‐NAD^+^ and BAD with IC_50_ values of 28 and 39 μM, respectively.

## Graphical abstract

**Modestly modified**: Three stable NAD^+^ analogues namely, c‐NAD^+^, S‐NAD^+^ and BAD were prepared by using an optimized chemoenzymatic procedure and were compared side‐by‐side for enzyme inhibition of Legionella effector enzyme SdeC, which is important in bacterial virulence. Minimal structural variation in the furanose ring or nicotinamide part of NAD^+^ leads to efficient inhibitors.

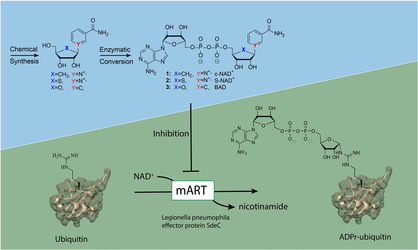

## Introduction

**Figure 1:**
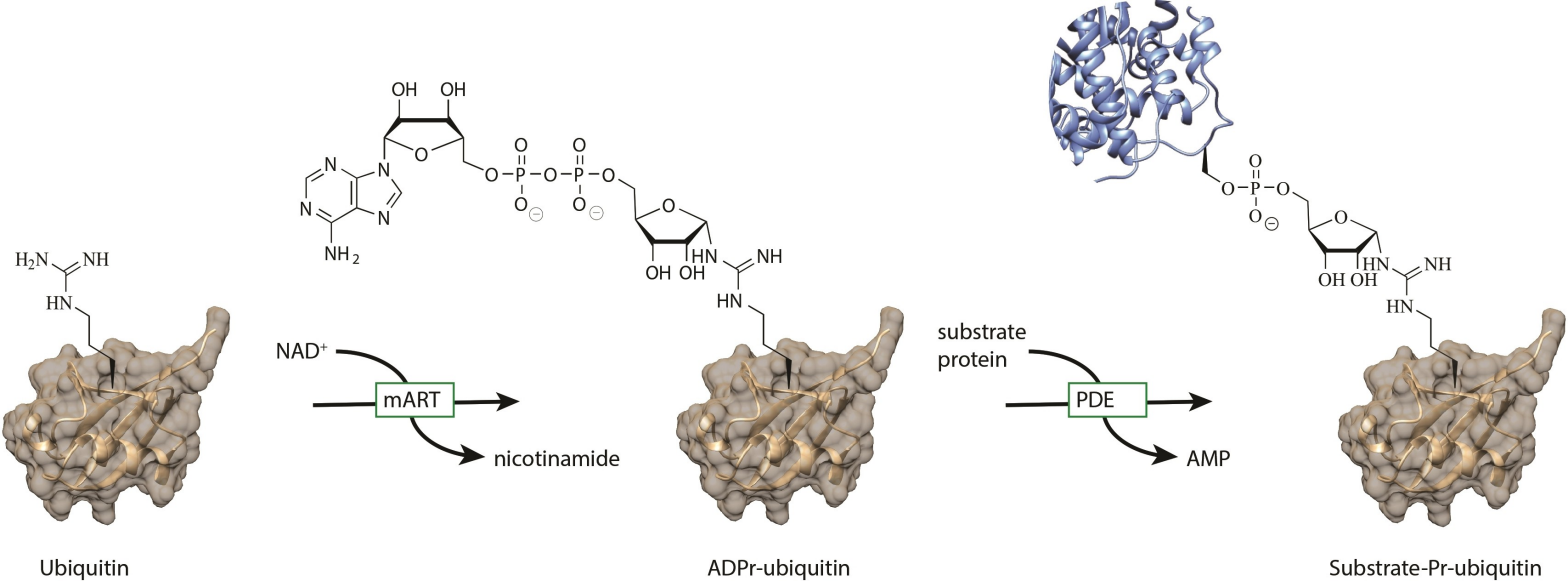
Schematic representation of mART and PDE action of SidE‐family enzymes on ubiquitin.

Many cellular proteins are decorated with post‐translational modifications (PTMs) during their life‐time that control their function, cellular localization and activity. One of these PTMs is adenosine‐diphosphate‐ribosylation (ADP‐ribosylation), in which a monoadenosine diphosphate ribosyl transferase enzyme (mART) transfers an ADPr fragment from an NAD^+^ molecule to the nucleophilic side chain of an amino acid residue of a target protein, releasing nicotinamide in the process. This mono ADPr‐modification can be elongated in a linear or branched fashion, giving rise to linear poly‐ and branched poly‐ADPr chains. Enzymes generating both the mono‐ADPr and poly‐ADPr modification play crucial roles in distinct biological processes.^1^ Another essential PTM that regulates many key cellular processes is ubiquitylation, in which a small 8.5 kDa protein called ubiquitin (Ub) is attached to the target protein.^2^ Installment of Ub is regulated by a cascade of three enzymes known as E1‐activing enzyme, E2‐conjugating enzyme and E3‐ligase. In this ATP‐dependent process the combination of specific E1‐E2‐E3 enzymes dictates the choice of substrate and site of ubiquitylation. Prokaryotes do not contain the Ub system, but a variety of bacterial pathogens do have the capability to use or hijack the host's Ub system.^3^ Recently, it was discovered that the *Legionella pneumophila* bacterium uses a family of so‐called SidE effector enzymes to mediate the ADP‐ribosylation of Ub as a crucial first step in ubiquitylating host proteins of their own choice, finally leading to Legionnaires disease in immunocompromised individuals. These SidE effectors contain multiple domains with different catalytic activities, including a mART and a phosphodiesterase (PDE) domain. Initially, the mART domain catalyzes the transfer of ADPr to arginine 42 (R42) of Ub, releasing nicotinamide. Subsequently the PDE domain will activate the phosphodiester bond, facilitating the covalent attachment to a serine residue of a host substrate protein while displacing adenosine mono‐phosphate (AMP; Figure 1).^4^, ^5^, ^6^, ^7^, ^8^ In this way, the enzyme is able to ubiquitylate the substrate, via a unique phosphoribosyl (Pr) linkage without the need for either E1‐E2‐E3 enzymes or ATP. Recent studies link this phenomenon of Pr ubiquitylation to the development and maintenance of a vacuole in which Legionella replicates, by modifying host proteins involved in ER fragmentation and membrane recruitment.^9^, ^10^ The unusual Pr ubiquitylation, with ADPribosylation of Ub as first step, is an interesting target for drug development, since this pathway seems to be Legionella specific as it has not been identified in mammals so far. Knocking down the SidE effectors severely effects bacterial virulence and hence these proteins are bona‐fide drug targets.^11^

## Results

**Figure 2:**
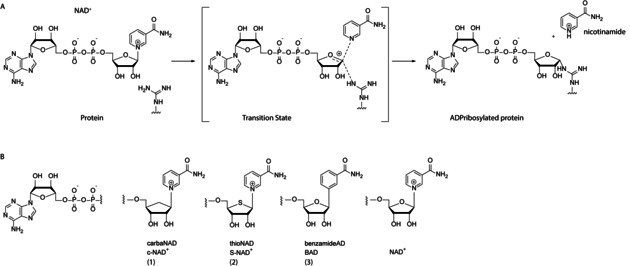
A) Reaction mechanism of ART mediated ADP‐ribosylation, B) NAD^+^‐based ART inhibitors **1**, **2** and **3**.

There are two distinct classes of ART enzymes, referred to as diphtheria‐toxin‐like or cholera‐toxin‐like, which have a HYE or RSE catalytic triad, respectively.^12^ Both types transfer ADPr in a reaction that proceeds through a transition state having a ribosyl oxocarbenium ion character (Figure 2A). Several NAD^+^ mimics that are unable to form such an oxocarbenium intermediate, such as carbanicotinamide adenosine dinucleotide (c‐NAD^+^, **1**), thionicotinamide adenosine dinucleotide (S‐NAD^+^, **2**) and benzamide adenosine dinucleotide (BAD, **3**; Figure 2B), have been developed as inhibitors of NAD^+^ consuming enzymes and have shown moderate to good inhibition.^13^, ^14^, ^15^ c‐NAD^+^ has been shown to inhibit activity of the cholera toxin A ART enzyme^16^ and NAD^+^‐consuming enzymes Sirt3/5,^17^ but was ineffective towards NAD^+^‐glycohydrolase. c‐NAD^+^ is also ineffective in inhibiting PARP1, while BAD is a low micromolar PARP1 inhibitor.^18^ S‐NAD^+^ was developed more recently and shows inhibition of NAD^+^‐dependent enzyme CD38.^19^

In this light, we decided to explore these three promising NAD^+^ analogues, c‐NAD^+^ (**1**), S‐NAD^+^ (**2**) and BAD (**3**), for their inhibitory potential on the ART function of the Legionella SidE family member SdeC. Fully synthetic approaches towards BAD and c‐NAD^+^ have been reported, but formation of the dinucleotide, through coupling of adenosine monophosphate and the phosphorylated ribose analogues remains troublesome. Therefore we decided to adopt a chemo‐enzymatic procedure, recently used to prepare S‐NAD^+^, to form the difficult pyrophosphate linkages in c‐NAD^+^ and BAD.^19^ We synthesized the carbanicotinamide riboside and benzamide riboside using optimized procedures from literature and developed a novel synthesis for the thioriboside. Subsequent chemo‐enzymatic conversion using nicotinate riboside kinase (NRK1) to produce the nicotinamide mononucleotides (NMNs) and subsequent action of nicotinamide mononucleotide adenylyl transferase (NMNAT1) would yield us the three NAD analogues c‐NAD^+^ (**1**), S‐NAD^+^ (**2**) and BAD (**3**).

**Scheme 1:**
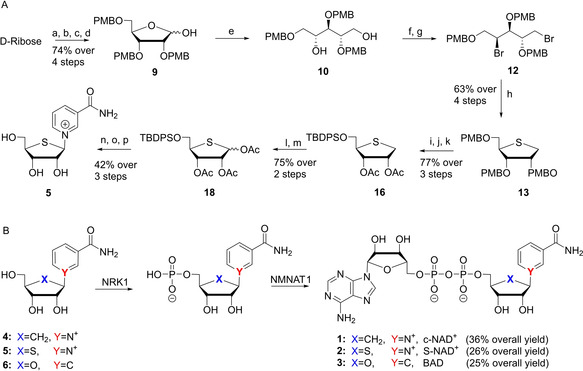
A) Synthesis of thionicotinamide riboside (**5**) a) AllOH, AcCl, 0 °C; b) PMBCl, NaH, DMF; c) KO*t*Bu, DMF, 110 °C; d) THF, NaHCO_3_ (sat. aq.), I_2_; e) NaBH_4_, MeOH; f) MsCl, Et_3_N, CH_2_Cl_2_; g) LiBr, MEK, 80 °C; h) Na_2_S, DMF, 100 °C; i) TFA, CH_2_Cl_2_; j) TBDPSCl, Im., CH_2_Cl_2_; k) Ac_2_O, pyr.; l) *m*‐CPBA, CH_2_Cl_2_, −40 °C; m) Ac_2_O, 100 °C; n) nicotinamide, BSTFA, TMSOTf, ACN, 80 °C; o) HF/pyr.; p) NH_3_/MeOH, 0 °C. B) enzymatic conversion of ribosides **4**–**6** towards NAD^+^ analogues **1**–**3**. PMB=*para*‐methoxybenzyl, TBDPS=*tert*‐butyl‐*di*‐phenyl silyl, NRK1=nicotinate riboside kinase 1, NMNAT1=nicotinamide mononucleotide adenylyl transferase 1.

The known carbanicotinamide riboside (**4**)^17^ and benzamide riboside (**6**;^14^, ^20^ Scheme 1B) were prepared following the optimized reported procedures. We devised a new synthetic route for the third analogue, thionicotinamide riboside (S‐NR, **5**) that was recently described.^19^ We opted to employ the synthesis depicted in Scheme 1A that is based on our prior studies on thioribosides.^21^ First, we synthesized protected thioribitol **13** from ribose, essentially as described by us and others.^21^, ^22^ In brief, the synthesis started with readily accessible and inexpensive D‐ribose that was first converted into lactol **9**, the aldehyde functionality of which was next reduced to give ribitol **10**. Mesylation and double bromide substitution then provided dibromide **12**, which was treated with sodium sulfide to give thioribitol **13**. After removal of the three *p*‐methoxybenzyl ethers by acidolysis, we selectively protected the primary alcohol with a TBDPS group and the secondary alcohols with acetyls to obtain **16**. The thioether was then oxidized to a sulfoxide using *m*‐CPBA at low temperature and with controlled amounts of the oxidizing agent to avoid an overoxidation to the corresponding sulfone. The subsequent Pummerer‐rearrangement was performed by dissolving the sulfoxide obtained from **16** in acetic anhydride and heating the reaction mixture to 120 °C. The rearrangement proceeded regio‐ and stereoselectively to provide thioribosyl donor **18** as a mixture of anomers. We observed that the Pummerer rearrangement of a per‐O‐acetylated thioribitol led to the formation of 4‐O‐acetyl thioribitol by oxidation at C‐4, and hence the electron donating properties of the silyl protection at C5 was essential in our synthesis. Donor **18** was next glycosylated with nicotinamide in a Vorbrüggen‐type glycosylation, using TMSOTf in acetonitrile, yielding a mixture of α/β‐anomers in 75 % yield. Finally, the silyl ether was removed using HF in pyridine, and the acetyls were removed using ammonia in methanol at 0 °C, at which stage the anomers could be separated by RP‐HPLC to provide thionicotinamide riboside **5**. Thus, S‐NR **5** was obtained in 16 steps in an overall yield of 3.6 %, which compares favorably with the method previously reported for the synthesis of this compound.^19^ With nicotinamides **4** and **5** and benzamide **6** in hand, we next focused on the enzymatic phosphorylation of the 5’‐hydroxy function using NRK1, followed by introduction of the pyrophosphate using NMNAT1 (Scheme 1B). The riboside was dissolved in buffer containing ATP, MgCl_2_ and DTT after which NRK1 was added to install the phosphate group on the 5’‐hydroxy function selectively. After one hour, complete formation of the nicotinamide mononucleotides was observed, and NMNAT1 was added to the reaction mixture to install AMP and form the respective NAD^+^ analogues. After reacting for 16 hours LCMS analysis revealed consumption of the mono‐nucleotides and formation of the NAD^+^‐analogues, that where purified using RP‐HPLC to yield us compounds **1**, **2** and **3** in multi‐milligram amounts (36, 26 and 25 % yield, respectively) as off‐white powders.

**Figure 3:**
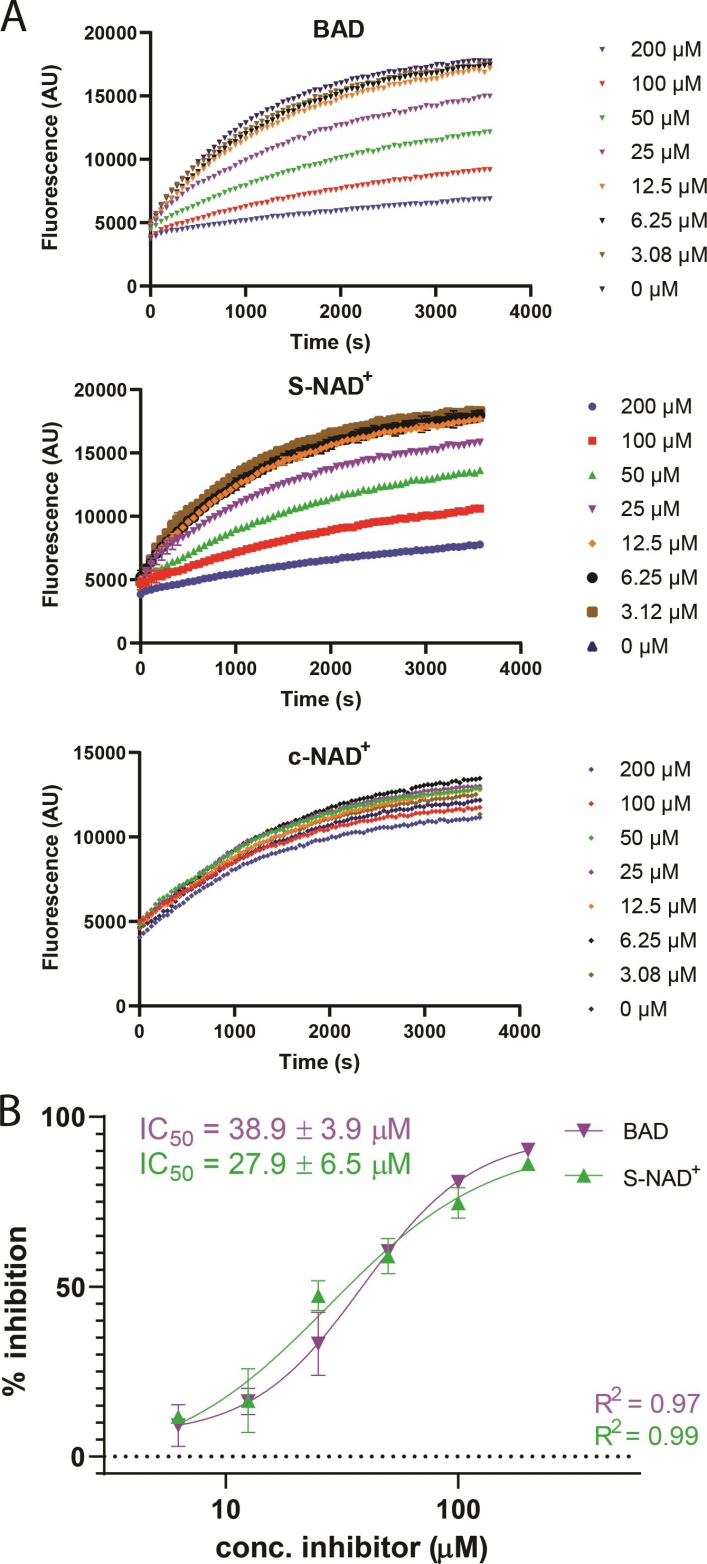
A) ϵ‐NAD^+^ hydrolysis assays showing nicotinamide displacement by SdeC ART activity and its concentration‐dependent inhibition by BAD, S‐NAD^+^ and c‐NAD^+^, B) IC_50_‐curves of S‐NAD^+^ and BAD.

Next we investigated inhibition of SdeC activity by c‐NAD^+^ (**1**), S‐NAD^+^ (**2**) and BAD (**3**) using an ϵ‐NAD^+^ hydrolysis assay.^23^ In ϵ‐NAD^+^, the fluorescence of etheno‐adenosine is quenched by nicotinamide and the formation of ADPr−Ub as a result of ART activity of SdeC releases nicotinamide, resulting in a fluorescent signal. The increase in fluorescence is therefore a direct measure of substrate ϵ‐NAD^+^‐consumption and enzyme activity. In this assay, Ub and ϵ‐NAD^+^ are incubated with SdeC and increase in fluorescence is measured over time. We first assessed the affinity of unmodified NAD^+^ by incubating SdeC with both ϵ‐NAD^+^ and NAD^+^. Such substrate competition between the two NAD^+^ species led to a concentration‐dependent decrease in fluorescent signal upon increasing the amount of NAD^+^, allowing us to determine the IC_50_ value of NAD^+^ for SdeC to be 27.7±1.9 μM (see extended data in Figure 3). When performing a similar experiment where c‐NAD^+^, S‐NAD^+^ or BAD were incubated at increasing concentrations with SdeC, Ub and ϵ‐NAD^+^, we observed concentration‐dependent inhibition of ART‐activity for S‐NAD^+^ and BAD. However, only a marginal effect for c‐NAD^+^ was detected in the used concentration range up to 200 μM (Figure 3A). IC_50_ values for BAD and S‐NAD^+^ were determined to be 27.9±6.5 and 38.9±3.9 μM, respectively (Figure 3B). S‐NAD^+^ thus is an inhibitor of SdeC with a comparable affinity as native substrate NAD^+^, whereas BAD is a good inhibitor with a slightly higher IC_50_.

## Conclusion

In conclusion, we have shown the improved chemical synthesis of thionicotinamide riboside and converted it alongside with carbanicotinamide riboside and benzamide riboside to the respective NAD^+^ analogues by using the enzyme combination NRK1 and NMNAT1. The adaptability of this chemoenzymatic approach, previously demonstrated only for S‐NAD^+^, allowed the fast and scalable (∼5 mg) construction of all three NAD^+^ analogues, including c‐NAD^+^ and BAD. Thus, we reveal that minimal structural variation in the furanose ring as well as modification of the nicotinamide part does not interfere with the activity of the NRK1/NMNAT1 enzymes. We then tested the three compounds on their inhibitory capacity towards pathogenic Legionella enzyme SdeC. S‐NAD^+^ and BAD, showed micromolar inhibition of the Legionella enzyme, with an affinity for the enzyme in the same order of magnitude as the native substrate NAD^+^. The carba analogue only minimally inhibited the ART activity of SdeC, indicating that the substitution of the oxygen atom in the furanose ring with methylene was not tolerated, probably due to its rigidity enhancing effect on the five‐membered ring. When comparing the IC_50_ values of S‐NAD^+^ (27.9 μM) and BAD (38.9 μM) with NAD^+^ (27.7 μM), the S‐NAD^+^ seems to be most comparable to the native substrate. To the best of our knowledge these are the first NAD^+^‐mimics that inhibit a member of the Legionella SidE family. Although the compounds tested here will most likely not be specific for the Legionella SidE family, as they also inhibit other NAD^+^‐consuming enzymes, they do form a starting point for obtaining selective inhibitors. A recent report shows that relatively minor modifications at the 3′‐OH of a modified NAD^+^ analogue greatly enhances selectivity towards specific enzymes.^24^ Based on the combined insight that both the thioribofuranose and benzamide scaffold in NAD^+^ mimics inhibits SdeC activity, and that 3’‐OH modification can tune specificity of ADPr transferases, potential selective inhibitors might be developed in the near future giving rise to a new class of antibiotic candidates against Legionella infection.

## Conflict of interest

The authors declare no conflict of interest.

## Supporting information

As a service to our authors and readers, this journal provides supporting information supplied by the authors. Such materials are peer reviewed and may be re‐organized for online delivery, but are not copy‐edited or typeset. Technical support issues arising from supporting information (other than missing files) should be addressed to the authors.

Supplementary
