# Supplementary material for: Synthesis of Stable NAD+ Mimics as Inhibitors for the Legionella pneumophila Phosphoribosyl Ubiquitylating Enzyme SdeC
Source: Chembiochem. 2020 Jun 16;21(20):2903–7. doi: 10.1002/cbic.202000230 (PMC7687180; doi:10.1002/cbic.202000230)

ChemBioChem

Supporting Information

Synthesis of Stable NAD$^+$ Mimics as Inhibitors for the *Legionella pneumophila* Phosphoribosyl Ubiquitylating Enzyme SdeC

Jerre M. Madern, Robbert Q. Kim, Mohit Misra, Ivan Dikic, Yong Zhang, Huib Ovaa[+], Jeroen D. C. Codée, Dmitri V. Filippov,* and Gerbrand J. van der Heden van Noort*

Supporting Information

General Procedures

General reagents were obtained from Sigma Aldrich, Fluka and Acros and used as received. Solvents were purchased from BIOSOLVE or Aldrich. Column chromatography was carried out on silica gel (0.035-0.070 mm, 90Å, Acros). Nuclear magnetic resonance spectra (^{1}H-NMR, ^{13}C-NMR (APT), ^{31}P-NMR) were determined using a Bruker AV-300 MHz, AV-400 MHz or AV-500 MHz at 298 K. ^{1}H-NMR spectra in water were measured using HDO presaturation as indicated. Multiplicities in NMR spectra are indicated with the symbols 'd' (doublet), 'dd' (double doublet), 's' (singlet), 't' (triplet) and 'm' (multiplet). Chemical shifts are given in ppm (δ) relative to TMS (0 ppm) or indirectly referenced to H_3PO_4 (0.00 ppm) in D_2O via the solvent residual signal.

***LC-MS measurements**:*

Waters 2795 Separation Module (Alliance HT) using a Phenomenex Kinetex C18-column (2.1x50, 2.6 µm), Waters 2996 Photodiode Array Detector (190-750 nm) and LCT™ ESI-Mass Spectrometer. Samples were run using 2 mobile phases: A = 1% CH_3CN, 0.1% formic acid in water and B = 1% water and 0.1% formic acid in CH_3CN. Flow rate= 0.8 mL/min, runtime= 6 min, column T= 40°C. Gradient: 0 - 95% B. Data processing was performed using Waters MassLynx Mass Spectrometry Software 4.1 (deconvolution with MaxEnt1 function).

RP-HPLC purifications

Shimadzu semi-preparative RP-HPLC system, equipped with a Waters C18-Xbridge 5 µm OBD (10 x 150 mm) column at a flowrate of 6.5 mL/min. using 2 mobile phases: A: MQ + 0.05% FA, B: CH_3CN + 0.05 % FA. Gradient: 0 -> 15% B.

HRMS-measurements

High resolution mass spectra were recorded on a Waters XEVO-G2 XS Q-TOF mass spectrometer equipped with an electrospray ion source in positive mode (source voltage 3.0 kV, desolvation gas flow 900 L/hr, temperature 250 °C) with resolution R = 22000 (mass range m/z = 50-2000) and 200 pg/uL Leu-Enk (m/z = 556.2771) as a "lock mass".

General enzymatic conversion of riboside to NAD$^+$-analogues

The appropriate riboside (7.5 mg, ~30 µmol) was dissolved in 750 µL buffer (20 mM TRIS, 50 mM NaCl) and supplemented with 250 mM ATP, 12 mM $MgCl_2$ and 2 µM DTT at pH 7.5. Subsequently NRK1 (20 µM final concentration) was added and incubated at room temperature for 60 min. after which LC-MS analysis revealed complete consumption of the riboside and formation of the NMN. NMNAT1 (2 µM final concentration) was added and incubated for 16 hours after which LC-MS analysis revealed complete consumption of the NMN and formation of the NAD$^+$-analogue. Purification using RP-HPLC (XBridge BEH C18 OBD column, 5 µm, 10 mm x 150 mm) and lyophilization of the appropriate fractions yielded the pure NAD$^+$-analogue as off-white powder.

c-NAD (1)

c-NAD was prepared by the general enzymatic conversion procedure as described above. Yield 6.63 mg (36%).^{31}**P NMR** (121 MHz, D_2O) δ -10.79, -10.96, -11.28, -11.45. ^{1}**H NMR** (300 MHz, D_2O, presat.) δ 9.25 (s, 1H), 9.03 (dt, *J* = 6.3, 1.4 Hz, 2H), 8.77 (dt, *J* = 8.2, 1.4 Hz, 1H), 8.54 (s, 1H), 8.48 (s, 2H), 8.28 (s, 2H), 8.09 (dd, *J* = 8.1, 6.2 Hz, 2H), 7.32 (d, *J* = 1.3 Hz, 2H), 6.01 (d, *J* = 5.4 Hz, 2H), 5.01 – 4.86 (m, 1H), 4.39 (dd, *J* = 5.3, 3.9 Hz, 2H), 4.26 (t, *J* = 3.1 Hz, 2H), 4.21 – 4.04 (m, 4H), 4.04 – 3.98 (m, 1H), 3.92 (dt, *J* = 10.0, 4.7 Hz, 2H), 2.32 (d, *J* = 5.1 Hz, 2H), 2.07 (ddd, *J* = 13.4, 10.6, 7.6 Hz, 2H). ^{13}**C NMR** (75 MHz, D_2O) δ 165.51, 149.88, 145.58, 145.00, 142.88, 133.95, 128.63, 87.88, 84.19, 84.07, 77.05, 75.88, 74.63, 72.24, 70.21, 66.71, 65.04, 59.27, 43.00, 38.67, 28.78. **HRMS**: $[C_{22}H_{30}N_7O_{13}P_2]^+$: found 662.1406, calc. 662.1377

S-NAD (2)

S-NAD was prepared by the general enzymatic conversion procedure as described above. Yield 5.82 mg (26%).^{31}**P NMR** (121 MHz, D_2O) δ -11.23, -11.40, -11.59, -11.76. ^{13}**C NMR** (75 MHz, D_2O δ 165.47, 149.81, 148.27, 145.84, 145.45, 142.81, 133.93, 128.71, 118.41, 87.81, 84.14, 84.02, 81.27, 81.00, 74.88, 74.56, 70.18, 66.06, 66.00, 65.03, 59.21, 52.70, 52.59. ^{1}**H NMR** (300 MHz, D_2O, presat.) δ 9.46 (s, 1H), 9.46 – 9.36 (m, 2H), 8.78 (dt, *J* = 8.1, 1.4 Hz, 3H), 8.48 (s, 4H), 8.28 (s, 4H), 8.13 (dd, *J* = 8.1, 6.2 Hz, 4H), 6.01 (dd, *J* = 6.0, 4.6 Hz, 7H), 4.69 – 4.56 (m, 1H), 4.62 – 4.49 (m, 1H), 4.44 – 4.31 (m, 5H), 4.33 (d, *J* = 2.6 Hz, 2H), 4.25 (tt, *J* = 8.0, 3.0 Hz, 5H), 4.25 – 4.12 (m, 2H), 4.17 – 4.00 (m, 9H), 3.64 (h, *J* = 3.5, 2.9 Hz, 4H). **HRMS**: $[C_{21}H_{28}N_7O_{13}P_2S]^+$ found 680.0976, calc. 680.0941

BAD (3)

BAD was prepared by the general enzymatic conversion procedure as described above. Yield 3.25 mg (25%). ^{31}**P NMR** (121 MHz, D_2O) δ -11.02, -11.19, -11.29, -11.46. ^{13}**C NMR** (75 MHz, D_2O) δ 171.92, 149.54, 147.99, 139.53, 132.42, 130.52, 128.96, 127.28, 125.00, 118.25, 87.88, 84.08, 83.97, 83.31, 83.20, 82.90, 77.20, 74.48, 71.47, 70.14, 65.10. ^{1}**H NMR** (300 MHz, D_2O, presat.) δ 8.40 (s, 1H, H8 Ade), 8.15 (s, 1H, H2 Ade), 7.56 (s, 1H, H2 benzamide), 7.48, 7.40 (2x d, *J* = 7.7, 1.5 Hz, 2H, H4 benzamide, H6 benzamide), 7.25 (t, *J* = 7.7 Hz, 1H, H5 benzamide), 5.92 (d, *J* = 5.3 Hz, 1H, H1"), 4.59 (t, *J* = 5.2 Hz, 1H, H2"), 4.34 (t, *J* = 5.1, 1H, H3"), 4.22 (m, *J* = 3.2, 2.8 Hz, 1H, H4"), 4.13 (dd, *J* = 5.1, 3.0 Hz, 1H), 4.05 (td, *J* = 6.1, 5.2, 3.2 Hz, 2H), 3.91 (dd, *J* = 7.4, 5.2 Hz, 1H). **HRMS**: $[C_{22}H_{28}N_6O_{14}P_2 + H]^+$: found 663.1202, calc. 663.1217

Chemical synthesis:

c-NR (4)

Carba-nicotinamide riboside was prepared following the reported procedure of Szczepankiewicz.[1]

^{1}**H NMR** (300 MHz, D_2O, presat.) δ 9.34 (d, *J* = 1.6 Hz, 1H, H2), 9.09 (dt, *J* = 6.4, 1.3 Hz, 1H, H6), 8.88 (dt, *J* = 8.1, 1.4 Hz, 1H, H4), 8.19 (dd, *J* = 8.1, 6.2 Hz, 1H, H5), 5.03 (ddd, *J* = 10.9, 9.5, 7.8 Hz, 1H, H1'), 4.38 (dd, *J* = 9.4, 5.9 Hz, 1H, H2'), 4.05 (dd, *J* = 5.9, 3.1 Hz, 1H, H3'), 3.68 (d, *J* = 5.9 Hz, 2H, H5'), 2.64 (dt, *J* = 13.2, 8.2 Hz, 1H, H6' cis), 2.28 (ddt, *J* = 8.7, 5.9, 2.9 Hz, 1H, H4'), 1.93 (ddd, *J* = 13.0, 11.0, 8.6 Hz, 1H, H6' trans). ^{13}**C NMR** (75 MHz, D_2O) δ 165.70 ($CONH_2$ C7), 145.37 (C6), 144.77 (C4), 143.10 (C2), 134.10 (C3), 128.64 (C5), 76.71 (C2'), 75.60 (C1'), 71.56 (C3'), 62.62 (C5'), 44.69 (C4'), 29,24 (C6'). **HRMS**: $[C_{12}H_{17}N_2O_4]^+$ 253.1152 found, 253.1188 calc.

S-NR (5)

Thio-nicotinamide riboside was prepared following the procedure reported below.

B-R (6)

Benzamide riboside was prepared following the reported procedures of Krohn[2]. The final debenzylation reaction was performed following the procedure reported by Bonnac[3].

^{1}**H NMR** (300 MHz, Methanol-d_4) δ 8.00 (d, *J* = 1.8 Hz, 1H, H2-NA), 7.84 (dt, *J* = 7.8, 1.5 Hz, 1H, H6-NA), 7.68 (dt, *J* = 7.8, 1.4 Hz, 1H, H4-NA)), 7.48 (t, *J* = 7.7 Hz, 1H, H5-NA), 4.81 (d, *J* = 7.0 Hz, 1H, H1), 4.15 (dd, *J* = 5.7, 3.8 Hz, 1H, H3), 4.05 (dt, *J* = 4.7, 3.6 Hz, 1H, H4), 3.93 (dd, *J* = 7.0, 5.6 Hz, 1H, H2), 3.82 (qd, *J* = 12.0, 4.1 Hz, 2H, H5). ^{13}**C NMR** (75 MHz, Methanol-d_4) δ 171.00 ($CONH_2$), 141.05(C1-NA),

133.37 (C3-NA), 129.75 (C4-NA), 128.20 (C5-NA), 126.72 (C6-NA), 125.12 (C2-NA), 85.27 (C4), 83.47 (C1), 77.83 (C2), 71.65 (C3), 62.07 (C5). **HRMS**: $[C_{12}H_{15}NO_5 + H]^+$ 254.1068 found, 254.1028 calc.

Chemical synthesis of S-NR:

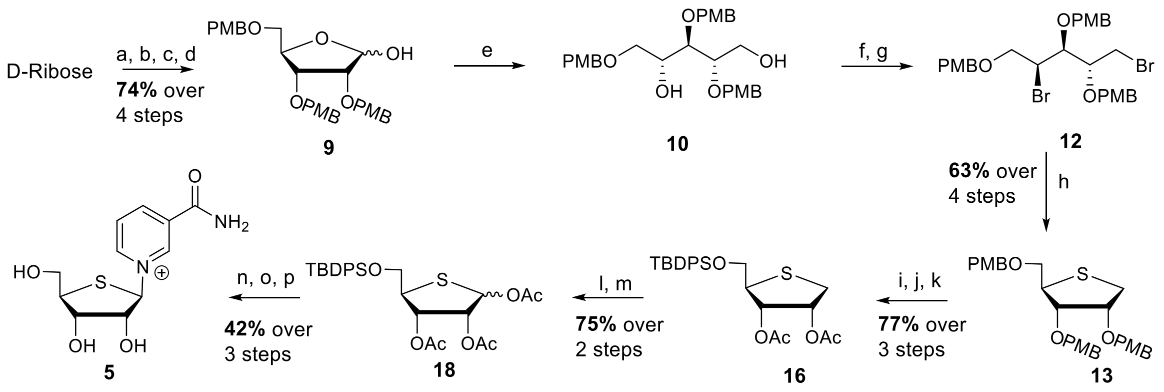

1-O-Allyl-α,β-D-ribofuranoside (7)

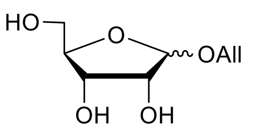

D-ribose (200 mmol, 30.0 g) was suspended in allyl alcohol (500 ml). The suspension was cooled to 0 °C and acetyl chloride (60 mmol, 4.3 ml) was slowly added. The reaction mixture was stirred for 5 additional hours. The reaction was quenched by the addition sodium bicarbonate until pH 7. The reaction mixture was filtered, concentrated *in vacuo* and used in the next step without purification.

1-O-Allyl-2,3,5-tri-O-paramethoxybenzyl-α,β-D-ribofuranoside (8)

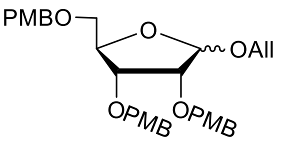

Crude **7** (200 mmol), dried by coevaporating with dry dioxane and toluene, was dissolved in DMF (500 ml). The solution was cooled to 0 °C and sodium hydride (700 mmol, 28.0 g, 60% dispersion in mineral oil) was slowly added. After hydrogen gas formation stopped paramethoxybenzyl chloride (640 mmol, 100.2 ml) was slowly added and the reaction mixture was stirred at room temperature for 16 hours. The reaction was quenched by the addition of ice. The reaction mixture was diluted with diethylether and washed with water, brine, dried ($MgSO_4$), concentrated *in vacuo* and used in the next step without purification.

2,3,5-tri-O-paramethoxybenzyl-α,β-D-ribose (9)

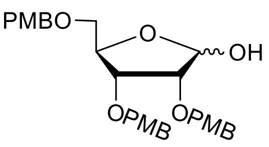

Crude **8** (200 mmol) and potassium tert-butoxide (300 mmol, 33.7 g) were dissolved in DMF (500 ml). The solution was stirred on 110 °C for 2 hours.

The reaction mixture was diluted by the addition of diethyl ether and the organic layer was extracted with water, brine, concentrated and the residue was dissolved in THF (250 ml) and aq. $NaHCO_3$ (sat.) (250 ml). Iodine (200 mmol, 50.5 g) was added and the reaction mixture was stirred for 1 hour. The reaction mixture was diluted with diethyl ether and washed with aq. $Na_2S_2O_3$ (sat.). The organic layer was dried ($MgSO_4$) and concentrated *in vacuo*. Column chromatography (Pentane: EtOAc, 90/10 – 50/50) yielded the title compound (147.5 mmol, 75.3 g, 74% over four steps). Analytical data was in agreement with literature.[4]

(2S,3R,4R)-2,3,5-tri-O-paramethoxybenzyl-pentane-1,4-diol (10)

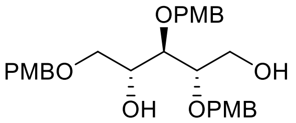

Compound **9** (141 mmol, 72.0 g) was dissolved in methanol (700 ml) and sodium borohydride (282 mmol, 10.7 g) was added in small portions over a period of 30 minutes at 0 °C. The reaction was then stirred for 1.5 hour at room temperature after which the solvent was removed *in vacuo* and the residue was dissolved in EtOAc and washed with water. The organic layer was dried ($MgSO_4$), concentrated *in vacuo* and used in the next step without purification.

(2S,3R,4R)-2,3,5-tri-O-paramethoxybenzyl-pentane-1,4-dimesylate (11)

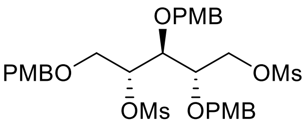

Crude **10** (141 mmol) was dissolved in DCM (700 ml) and triethylamine (564 mmol, 78.2 ml) was added. Mesyl chloride (423 mmol, 32.7 ml) was slowly added at -20 °C and the reaction was stirred for 30 minutes after which it was quenched upon the addition of ice. The organic layer was washed with water, aq. $NaHCO_3$ (sat.), dried ($MgSO_4$), concentrated *in vacuo* and used in the next step without purification.

(2S,3R,4S)-2,3,5-O-tri-paramethoxybenzyl-1,4-dibromopentane (12)

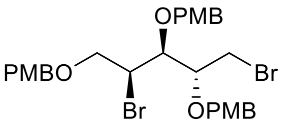

Crude **11** (141 mmol), co-evaporated with toluene to remove traces of water, was dissolved in dry 2-butanone (250 ml). Lithium bromide (1410 mmol, 122.5 g) was added and the reaction was refluxed for 20 hours. The reaction mixture was diluted with EtOAc and washed with water, brine, dried ($MgSO_4$), concentrated *in vacuo* and used in the next step without purification.

1,4-Anhydro-2,3,5-O-tri-paramethoxybenzyl-4-thio-D-ribitol (13)

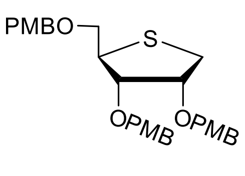

Crude **12** (141 mmol) and sodium sulfide nonahydrate (169 mmol, 40.6 g) were dissolved in DMF (600 ml). The reaction mixture was stirred at 100 °C for 60 minutes. The residue was dissolved in EtOAc washed with water, brine,

dried ($MgSO_4$) and concentrated *in vacuo*. Column chromatography (Pentane/ EtOAc, 98/2 – 80/20) yielded the title compound and the L-lyxitol derivative as a mixture (89.2 mmol, 45.6 g, 63% yield over four steps, D-ribitol:L-lyxitol 5.3:1). Crystallization with EtOAc and diethyl ether yielded the title compound as colorless crystals (61 mmol, 31.2 g), repeated column chromatography of the filtrate (Pentane/EtOAc, 98/2 – 80/20) yielded more title compound (14 mmol, 7.1 g). **^{1}H NMR** (400 MHz, $CDCl_3$) δ: 7.22 (m, 6H), 6.92 – 6.78 (m, 6H), 4.51 (s, 2H), 4.44 (dd, *J* = 11.4, 3.2 Hz, 4H), 3.97 (ddd, *J* = 6.9, 5.3, 3.5 Hz, 1H), 3.89 (dd, *J* = 4.4, 3.5 Hz, 1H), 3.79 (ds, 9H), 3.63 (td, *J* = 6.4, 4.4 Hz, 1H), 3.49 – 3.36 (m, 2H), 2.99 (AB, *J* = 10.6, 6.9 Hz, 1H), 2.84 (AB, *J* = 10.6, 5.4 Hz, 1H). **^{13}C NMR** (101 MHz, $CDCl_3$) δ: 159.3, 159.3, 130.3, 130.2, 129.7, 129.4, 129.4, 113.8, 113.8, 113.8, 80.5, 79.3, 72.8, 71.7, 71.5, 71.4, 55.4, 55.3, 47.4, 30.8. **IR**: 2996, 2935, 2906, 2857, 2835, 1612, 1586, 1511, 1302, 1243, 1099, 1032. **HRMS** $[C_{29}H_{34}O_6S + Na]^+$: 533.1962 found, 533.1968 calc. Analytical data was in agreement with literature.[21]

1,4-Anhydro-4-thio-D-ribitol (14)

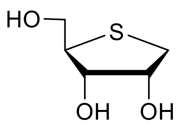

Compound **13** (55 mmol, 28.1 g) was dissolved in DCM (220 ml). TFA (55 ml) was added and the solution was stirred for 3 hours. The reaction mixture was diluted by adding toluene (300 ml) and it was concentrated *in vacuo*. Column chromatography (DCM: MeOH, 100/0 – 50/50) yielded the title compound (49.8 mmol, 7.48 g, 91%). **^{1}H NMR** (400 MHz, MeOD) δ: 4.23 (td, *J* = 5.2, 3.5 Hz, 1H), 3.95 (dd, *J* = 5.6, 3.5 Hz, 1H), 3.73 (dd, *J* = 11.2, 5.9 Hz, 1H), 3.56 (dd, *J* = 11.3, 6.5 Hz, 1H), 3.35 (m, 1H), 2.92 (dd, *J* = 10.8, 5.2 Hz, 1H), 2.76 (dd, *J* = 10.9, 5.3 Hz, 1H). **^{13}C NMR** (101 MHz, MeOD) δ: 77.7, 75.8, 65.5, 52.7, 33.5. **HRMS** $[C_5H_{10}O_3S+ + H]^+$: 151.0354 found, 151.0423 calc. Analytical data was in agreement with literature.[4]

1,4-Anhydro-5-O-tert-butyldiphenylsilyl-4-thio-D-ribitol (15)

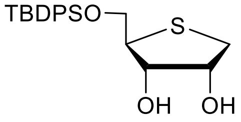

Compound **14** (19.8 mmol, 2.98 g) and imidazole (29.8 mmol, 2.03 g), dried by coevaporating with toluene, were dissolved in DCM (50 ml) and TBDPSCl (17.8 mmol, 4.63 ml) was slowly added. Then the reaction was stirred for 1 hour, quenched by the addition of water. The organic layer was then washed with water, dried ($MgSO_4$) and concentrated *in vacuo*. Column chromatography (Pentane: EtOAc, 90/10 – 50/50) yielded the title compound (15.8 mmol, 7.74 g, 89% based on TBDPSCl). **^{1}H NMR** (400 MHz, $CDCl_3$) δ: 7.66 (td, *J* = 7.8, 1.6 Hz, 4H), 7.50 – 7.36 (m, 6H), 4.42 – 4.35 (m, 1H), 4.17 – 4.09 (m, 1H), 3.88 (dd, *J* = 10.1, 5.2 Hz, 1H), 3.77 (dd, *J* = 10.0, 8.9 Hz, 1H), 3.51 (ddd, *J* = 8.9, 7.0, 5.2 Hz, 1H), 3.11 (d, *J* = 3.3 Hz, 1H), 3.04 (dd, *J* = 11.7, 4.6 Hz, 1H), 2.85 (dd, *J* = 11.7, 3.0 Hz, 1H), 2.52 (d, *J* = 3.7 Hz, 1H), 1.07 (s, 9H). **^{13}C NMR**

(101 MHz, $CDCl_3$) δ 135.7, 135.7, 132.7, 130.2, 128.1, 80.5, 77.5, 77.2, 76.8, 75.1, 67.2, 49.2, 33.7, 27.0, 19.3. **HRMS** $[C_{53}H_{64}O_3SSi + Na]^+$: 411.1424 found, 411.1421 calc.

1,4-Anhydro-2,3-bis-O-acetyl-5-O-tert-butyldiphenylsilyl-4-thio-D-ribitol (16)

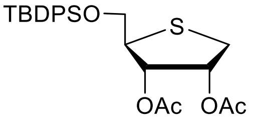

Compound **15** (16.5 mmol, 6.41 g) was dissolved in pyridine (100 ml) and acetic anhydride (65 ml). The reaction mixture was stirred on room temperature for 1 hour. The reaction mixture was concentrated *in vacuo*. Column chromatography (Pentane: EtOAc, 99/1 – 80/20) yielded the title compound (15.7 mmol, 7.43 g, 95%). ^{1}**H NMR** (500 MHz, $CDCl_3$) δ: 7.73 – 7.65 (m, 4H), 7.45 – 7.35 (m, 6H), 5.46 – 5.40 (m, 2H), 3.79 – 3.68 (m, 2H), 3.55 (q, J = 5.6 Hz, 1H), 3.15 – 3.07 (AB, 1H), 2.96 – 2.88 (AB, 1H), 2.06 (s, 3H), 2.04 (s, 3H), 1.07 (s, 9H). ^{13}**C NMR** (126 MHz, $CDCl_3$) δ: 167.0, 169.8, 135.6, 135.6, 133.0, 132.9, 129.8, 127.7, 127.7, 75.2, 73.8, 65.1, 49.4, 30.6, 26.7, 20.7, 19.2. **IR**: 3071, 3051, 2958, 2932, 2859, 1745, 1427, 1372, 1238, 1218, 1111, 1057, 1026. **HRMS** $[C_{25}H_{32}O_5SSi + Na]^+$: 495.1626 found, 495.1632 calc.

5-O-tert-butyldiphenylsilyl-1,4-anhydro-2,3-bis-O-acetyl-4-sulfoxide-D-ribitol (17)

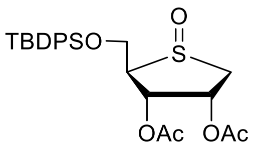

Compound **16** (15.5 mmol, 7.33 g) was dissolved in DCM (100 ml) and m-CPBA (17.0 mmol, 2.93 g) was added at -40 °C. The reaction mixture was stirred for 1 hour. The reaction mixture was quenched by the addition of aq. $Na_2S_2O_3$ (sat.) and the organic layer was washed with aq. $NaHCO_3$ (sat.). The organic layer was dried ($MgSO_4$), concentrated *in vacuo* and used in the next step without purification.

1,2,3-tri-O-Acetyl-5-O-tert-butyldiphenylsilyl-4-thio-α,β-D-ribofuranoside (18)

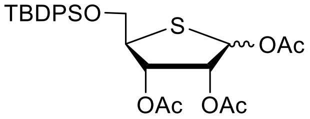

Crude **17** (15.5 mmol) was dissolved in acetic anhydride (100 ml) and stirred at 100 °C for 2 hours. The reaction mixture was concentrated *in vacuo*. Column chromatography (Pentane: EtOAc, 95/5 – 80/20) yielded the title compound (11.7 mmol, 6.2 g, 75% over two steps). ^{1}**H NMR** (400 MHz, $CDCl_3$, α-anomer) δ: 7.68 (ddt, J = 9.3, 6.3, 1.7 Hz, 4H), 7.47 – 7.35 (m, 6H), 6.27 (d, J = 5.0 Hz, 1H), 5.62 (dd, J = 4.5, 1.8 Hz, 1H), 5.45 (t, J = 4.7 Hz, 1H), 3.75 (AB, J = 7.5, 5.8 Hz, 1H), 3.70 – 3.61 (m, 2H), 2.11 (ds, 6H), 2.08 (s, 3H), 1.07 (s, 9H). ^{13}**C NMR** (101 MHz, $CDCl_3$, α-anomer) δ: 170.1, 167.0, 169.5, 135.8, 135.7, 132.8,

132.6, 123.0, 127.9, 77.3, 74.4, 73.5, 64.9, 51.1, 26.8, 21.1, 21.0, 20.6, 19.2. **^{1}H NMR** (400 MHz, $CDCl_3$, β-anomer) δ: 7.70 – 7.63 (m, 4H), 7.47 – 7.35 (m, 6H), 5.85 (d, *J* = 2.2 Hz, 1H), 5.62 (dd, *J* = 3.7, 2.2 Hz, 1H), 5.51 (dd, *J* = 7.9, 3.7 Hz, 1H), 3.84 – 3.72 (m, 2H), 3.72 – 3.63 (m, 1H), 2.13 (s, 3H), 2.07 (s, 3H), 2.01 (s, 3H), 1.06 (s, 9H). **^{13}C NMR** (101 MHz, $CDCl_3$, β-anomer) δ: 170.0, 169.8, 169.7, 137.5, 135.8, 135.7, 135.4, 135.4, 133.0, 133.0, 130.3, 130.3, 130.0, 129.9, 129.9, 128.0, 128.0, 127.9, 127.8, 127.8, 79.9, 76.3, 73.5, 63.8, 49.5, 26.8, 21.1, 20.9, 20.8, 19.4. **IR**: 3073, 2954, 2932, 2894, 2859, 1740, 1427, 1372, 1247, 1214, 1110, 1105, 1076, 1038, 1016. **HRMS** $[C_{27}H_{34}O_7SSi + Na]^+$: 553.1685 found, 553.1687 calc.

2,3-tri-O-acetyl-5-O-tert-butyldiphenylsilyl-4-thio-α,β-D-nicotinamide ribose (19)

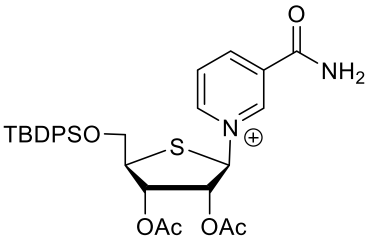

Compound **18** (1.28 mmol, 0.68 g) and nicotinamide (1.92 mmol, 0.26 g), co-evaporated with toluene to remove traces of water, was dissolved in ACN (13 ml) and BSTFA (3.84 mmol, 1.03 ml) was added. After stirring for 1 hour TMSOTf (1.28 mmol, 0.23 ml). The reaction mixture was refluxed for 2 hours after which pyridine (1 ml) was added to quench the reaction. The reaction mixture was concentrated *in vacuo*. Column chromatography (DCM:MeOH; 95/5 – 50/50) yielded the title compound (0.96 mmol, 0.63 g, 75%) as an inseparable anomeric mixture (α:β, 2.7:1). **^{1}H NMR** (400 MHz, MeOD) δ 9.82 (t, *J* = 1.6 Hz, 1H), 9.67 (t, *J* = 1.7 Hz, 1H), 9.57 – 9.48 (m, 1H), 9.12 (dt, *J* = 8.1, 1.4 Hz, 1H), 8.97 (dt, *J* = 8.2, 1.3 Hz, 1H), 8.58 (d, *J* = 5.0 Hz, 1H), 7.88 – 7.77 (m, 2H), 7.81 – 7.71 (m, 5H), 7.53 – 7.41 (m, 11H), 6.81 (d, *J* = 6.3 Hz, 1H), 6.61 (d, *J* = 6.0 Hz, 1H), 6.01 (dd, *J* = 6.4, 4.2 Hz, 1H), 5.83 – 5.71 (m, 2H), 4.18 (qd, *J* = 11.3, 4.7 Hz, 2H), 4.00 (dd, *J* = 11.0, 4.7 Hz, 1H), 3.92 (q, *J* = 4.5 Hz, 1H), 3.84 (dd, *J* = 11.0, 5.1 Hz, 1H), 2.16 (s, 3H), 2.11 (s, 3H), 2.02 (s, 1H), 1.97 (s, 1H), 1.84 (s, 1H), 1.15 (s, 9H), 1.13 (s, 4H). **^{13}C NMR** (101 MHz, MeOD) δ 151.4, 148.1, 136.0, 135.5, 135.4, 135.4, 130.1, 130.0, 129.9, 128.1, 127.9, 127.8, 127.7, 123.8, 78.9, 77.7, 73.3, 64.1, 52.1, 48.5, 48.3, 48.0, 47.8, 47.6, 47.4, 47.2, 47.0, 26.1, 25.9, 19.2, 18.9. **HRMS** $[C_{31}H_{37}N_2O_6SSi]^+$: 593.2129 found, 593.2136 calc.

2,3-tri-O-acetyl-4-thio-α,β-D-nicotinamide ribose (20)

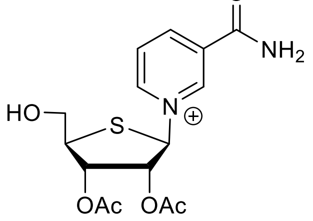

Compound **19** (0.96 mmol, 0.63 g), co-evaporated with pyridine to remove traces of water, was dissolved in pyridine (13 ml) and HF/pyr (1.30 ml) was added. The reaction mixture was stirred for 1 hour after which it was concentrated *in vacuo.* Column chromatography (DCM : MeOH; 95/5 – 50/50) yielded the title compound (1.29 g, unable to

determine yield due to salts) as an inseparable anomeric mixture. **^{1}H NMR** (600 MHz, MeOD) δ: 9.86 (s, 1H), 9.67 (s, 1H), 9.58 (d, *J* = 4.2 Hz, 1H), 9.44 – 9.32 (m, 1H), 8.97 (dd, *J* = 34.4, 5.7 Hz, 3H), 8.59 (s, 3H), 8.19 (d, *J* = 7.6 Hz, 3H), 8.06 (t, *J* = 7.5 Hz, 2H), 7.59 (s, 3H), 7.45 (s, 1H), 6.44 (d, *J* = 6.0 Hz, 1H), 5.89 – 5.76 (m, 2H), 5.57 – 5.48 (m, 2H), 3.96 (dd, *J* = 11.4, 2.6 Hz, 2H), 3.82 (dd, *J* = 11.6, 3.3 Hz, 2H), 3.74 (d, *J* = 2.8 Hz, 2H), 3.68 (dd, *J* = 11.6, 5.0 Hz, 1H), 2.05 (s, 3H), 1.94 (s, 3H), 1.81 (s, 1H), 1.72 (s, 2H). **^{13}C NMR** (150 MHz, MeOD) δ: 171.8, 171.4, 171.4, 170.5, 169.9, 165.0, 146.9, 146.8, 146.6, 145.5, 136.3, 134.7, 129.7, 128.3, 81.4, 80.2, 78.8, 76.7, 76.3, 75.3, 63.9, 63.1, 54.7, 54.5, 20.5, 20.3, 20.2, 20.0. **IR**: 3389, 3083, 2934, 2413, 1746, 1678, 1628, 1541, 1412, 1375, 1234, 1053, 1032. **HRMS** $[C_{15}H_{19}N_2O_6S]^+$: 355.0959 found, 355.0958 calc.

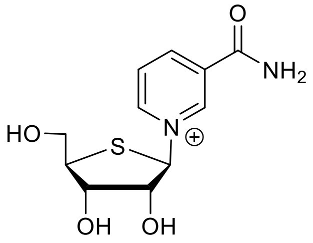

4-thio-β-D-nicotinamide ribose (5)

Crude **20** (0.96 mmol) was dissolved in 1M NH_3/MeOH (13 ml) The reaction mixture was stirred for 2 hours at 0 °C after which it was diluted with methanol and concentrated *in vacuo.* Column chromatography (ACN: H_2O, 100/0 – 30/70) yielded the title compound (0.51 mmol, 0.167 g, 56% over two steps) as an anomeric mixture. Further purification using HPLC separated the β-anomer (70 mg, 0.20 mmol) and α-anomer (51 mg, 0.16 mmol).

β-anomer: **^{1}H NMR** (300 MHz, D_2O, presat.) δ 9.63 (t, *J* = 1.6 Hz, 1H), 9.30 (dt, *J* = 6.3, 1.4 Hz, 1H), 8.81 (dt, *J* = 8.1, 1.4 Hz, 1H), 8.11 (dd, *J* = 8.1, 6.3 Hz, 1H), 6.04 (d, *J* = 5.8 Hz, 1H), 4.46 (dd, *J* = 5.8, 3.7 Hz, 1 H), 4.23 (t, *J* = 3.7 Hz, 1H), 3.94 – 3.74 (m, 2H), 3.54 (td, *J* = 4.6, 3.7 Hz, 1H). **^{13}C NMR** (75 MHz, D_2O) δ 145.5, 143.2, 134.1, 128.5, 81.0, 80.6, 73.9, 61.6, 53.9. **HRMS** $[C_{11}H_{15}N_2O_4S]^+$: 271.0769 found, 271.0747 calc.

α -anomer: **^{1}H NMR** (300 MHz, D_2O) δ 9.49 (t, *J* = 1.6 Hz 1H), 9.26 (dt, *J* = 6.2, 1.3 Hz, 1H), 8.81 (dt, *J* = 8.4, 1.6 Hz, 1H), 8.05 (dd, *J* = 8.0, 6.6 Hz, 1H), 6.31 (d, *J* = 6.0 Hz, 1H), 4.67 (1H), 4.26 (t, *J* = 3.5 Hz, 1H), 3.76 – 3.69 (m, 1H), 3.67 – 6.63 (m, 2H). **^{13}C NMR** (75 MHz, D_2O) δ 166.1, 146.9, 145.0, 144.4, 132.3, 126.7, 78.0, 75.1, 75.0, 62.4, 54.1.

Inhibition of NAD$^+$-analogues on SdeC activity (ε-NAD assay)

ADP-ribosylation of Ub and inhibition thereof by S-NAD, c-NAD and BAD was measured using an ε-NAD assay.[5] SdeC (10 nM) and Ub (50 µM) were treated with a concentration range of inhibitor (S-NAD, c-NAD or BAD) (200 nM, 100 nM, …, 6, 3, 0 nM) in buffer (50 mM HEPES/ 100 mM NaCl, pH 7.5) and the assay was started by the addition of ε-NAD (50 µM). The increase in fluorescence (λ_{ex} = 330

nm, λ_{em} = 410 nm) was monitored using a plate reader at 25 ^{o}C over time and the slope of the first 10 min. of linear increase was used to plot IC_{50}-curves. Experiments were performed in triplicates and shown graphs are mean values (depicted below).

Protein production

Expression plasmids

Generation of the plasmids for expression of NRK1 and NMNAT1 has been described before [Dai et. al. 2018]. In short, mouse full-length NRK1 and human full-length NMNAT1 were amplified with NcoI and XhoI restriction sites at the 5'- and 3'-end, using polymerase chain reaction (PCR). Fragments were then ligated into pET28a using NcoI and XhoI restriction enzymes and T4 ligase. Expression constructs were sequence-verified. The full-length SdeC effector was cloned in pGEX6P1 vector using the BamHI and XhoI restriction sites.

Protein production

NRK1 was expressed using BL21 (DE3) Rosetta2 pLysS cells in Terrific Broth, supplemented with 35 µg mL^{-1} chloramphenicol and 50 µg mL^{-1} kanamycin. Cells were grown at 37°C while shaking at 200 rpm. When the OD_{600} reached 1.5, expression was induced using 0.5 mM isopropyl ß-D-1-thiogalactopyranoside (IPTG), and the temperature was reduced to 18°C. The next day cultures were harvested by centrifugation at 4550 g (Beckman Coulter), the cell pellet was resuspended in His buffer (20 mM Tris pH 8.0, 200 mM NaCl and 20 mM imidazole) and frozen at -20°C or used directly for purification.

The NMNAT1 construct was transformed in BL21(DE3) cells and cultures were grown at 37°C in LB supplemented with 50 µg mL^{-1} kanamycin. Expression was induced when the cultures reached an OD_{600} between 0.6 and 0.8 using 0.5 mM IPTG and the cultures were left overnight shaking at 18°C. Cell pellets were harvested using centrifugation at 4550 g, resuspended in His buffer and frozen or used directly.

SdeC FL construct was transformed into T7 expressing *E. coli* chemically competent cells. Cultures were allowed to grow at 37 ^{o}C until O.D. was 0.6. Protein expression was induced by addition of 0.3 mM IPTG overnight at 18 ^{o}C. Cells were lysed in lysis buffer (50 mM Tris, 300 mM NaCl, 10% glycerol, 1 mM PMSF at pH 7.5).

Protein purification

Both NRK1 and NMNAT1 were purified using nickel affinity chromatography. The cells were lysed using sonication (QSonica) on ice using 20 cycles of 15” on and 45” off. The lysate was spun down at 21 kg (Beckman Coulter) for 45 minutes at 4°C. The supernatant was then applied to Ni-charged chelating sepharose beads (1 mL beads per 1L of expression volume; GE Healthcare) in a gravity flow column already equilibrated in His buffer. Beads were washed extensively (>100 CV), before eluting the protein using His buffer supplemented with 200 mM imidazole. Samples of all steps were analysed using SDS-PAGE and elution fractions containing the protein of interest (NRK1 ~27 kDa, NMNAT1 ~20 kDa) were pooled and set for overnight dialysis against 20 mM Tris pH8.0, 150 mM NaCl and 1 mM DTT at 4°C. Dialysate was then concentrated using spin filter concentrators (Amicon Millipore), aliquoted in 50 µL aliquots, flash frozen using liquid nitrogen and stored at -80°C. Protein concentrations were determined using the absorption at 280 nm, determined by NanoDrop (ThermoFisher) and the theoretical absorption coefficient (NRK1: 1.509; NMNAT1: 1.576).

SdeC were purified by loading the cleared lysate onto GST affinity beads and elution with 10 mM glutathione after washing the column. The obtained protein was further purified using the anion exchange Hiprep Q column. The protein was then purified using a size exclusion chromatography column in 10 mM HEPES, 150 mM NaCl, pH 7.5. Appropriate fractions were pooled, concentrated and flash frozen in liquid nitrogen and stored at – 80 °C until use.

Extended data:

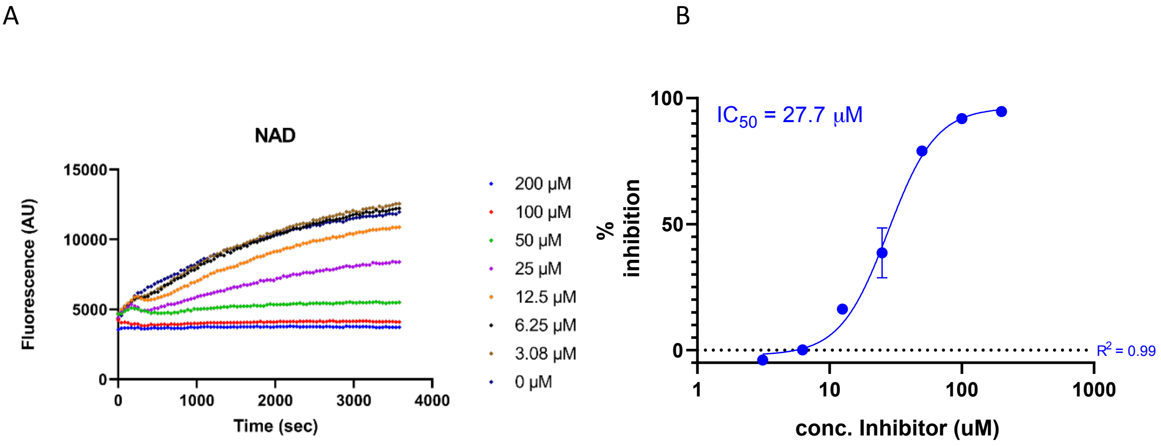

Extended Fig. 3. A) Competition assay between ε-NAD$^+$ and NAD$^+$ at varying concentrations of NAD$^+$, IC_{50} curve for NAD$^+$

References:

1. Szczepankiewicz, B.G., et al., *Synthesis of Carba-NAD and the Structures of Its Ternary Complexes with SIRT3 and SIRT5.* The Journal of Organic Chemistry, 2012. **77**(17): p. 7319-7329.
2. Krohn, K., H. Heins, and K. Wielckens, *Synthesis and cytotoxic activity of C-glycosidic nicotinamide riboside analogs.* Journal of Medicinal Chemistry, 1992. **35**(3): p. 511-517.
3. Bonnac, L.F., et al., *Efficient Synthesis of Benzamide Riboside, a Potential Anticancer Agent.* Nucleosides, Nucleotides & Nucleic Acids, 2007. **26**(10-12): p. 1249-1253.
4. Minakawa, N., et al., *An improved large scale synthesis of 1,4-anhydro-4-thio-D-ribitol.* Tetrahedron, 2003. **59**: p. 1699-1702.
5. Barrio, J.R., J.A. Secrist, 3rd, and N.J. Leonard, *A fluorescent analog of nicotinamide adenine dinucleotide.* Proceedings of the National Academy of Sciences of the United States of America, 1972. **69**(8): p. 2039-2042.

c-NAD (1)

^{1}H-NMR (300 MHz, D_2O)

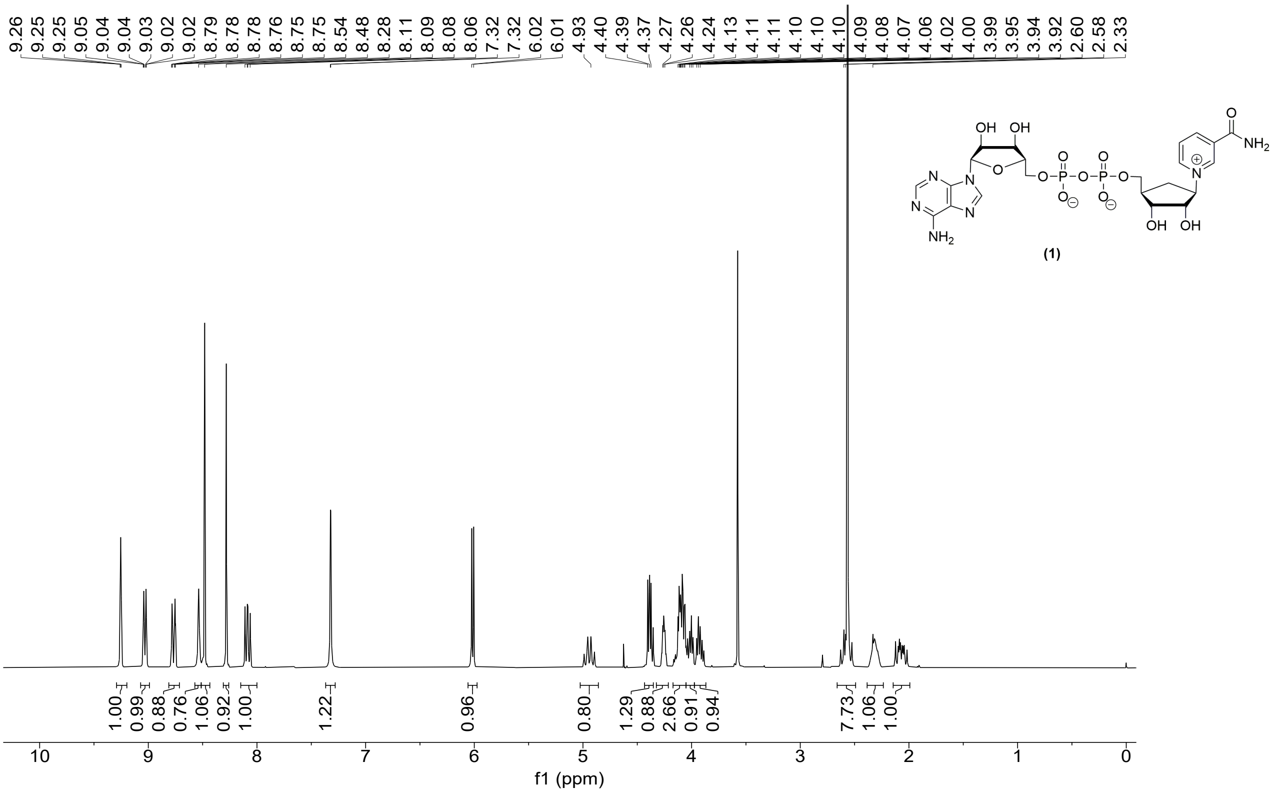

^{13}C-NMR (75 MHz, D_2O)

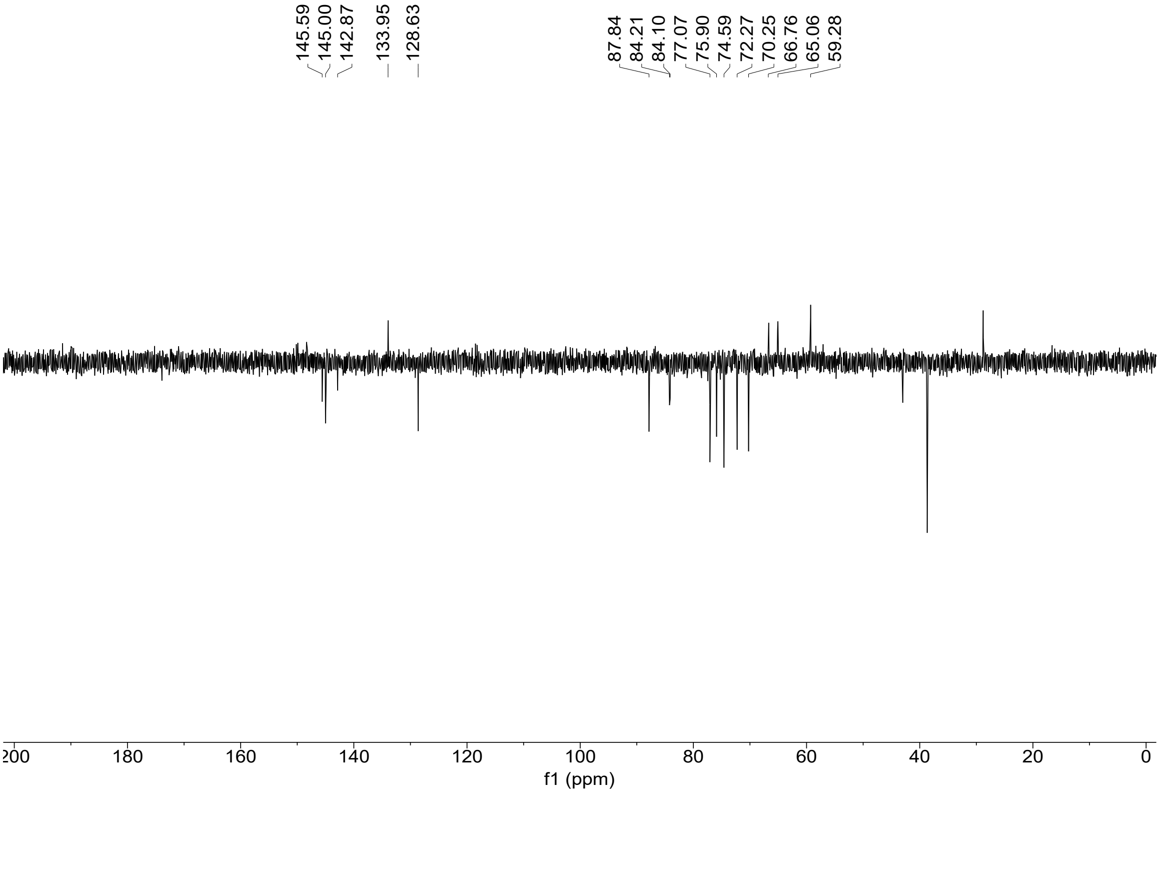

^{31}P-NMR (121 MHz, D_2O)

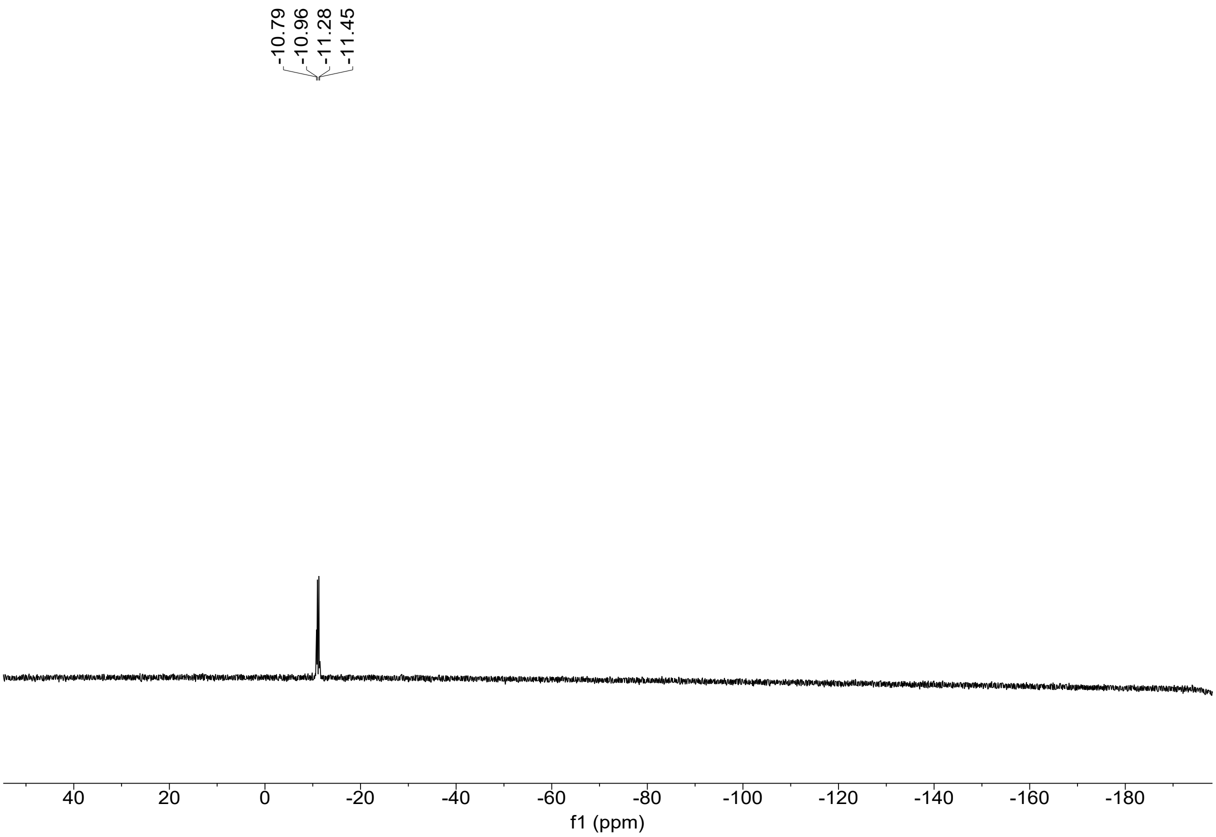

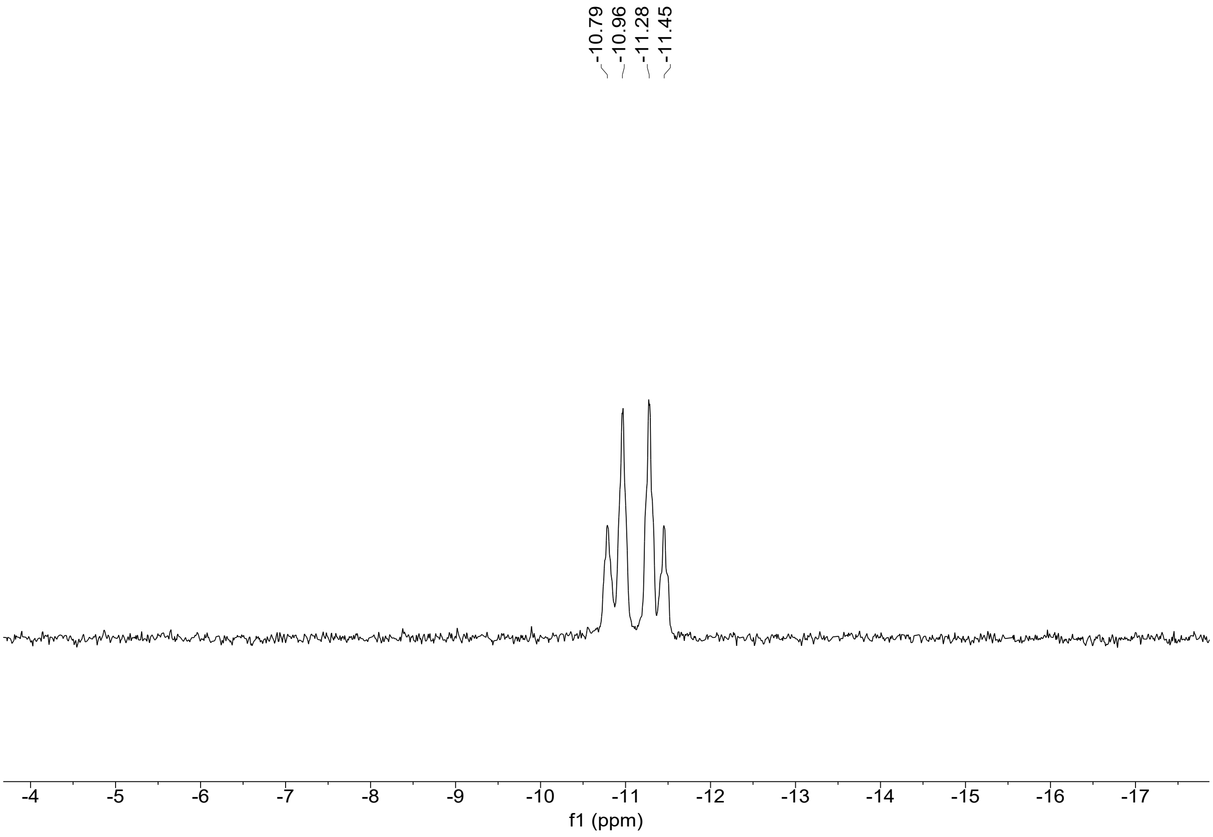

S-NAD (2)

^{1}H-NMR (300 MHz, D_2O)

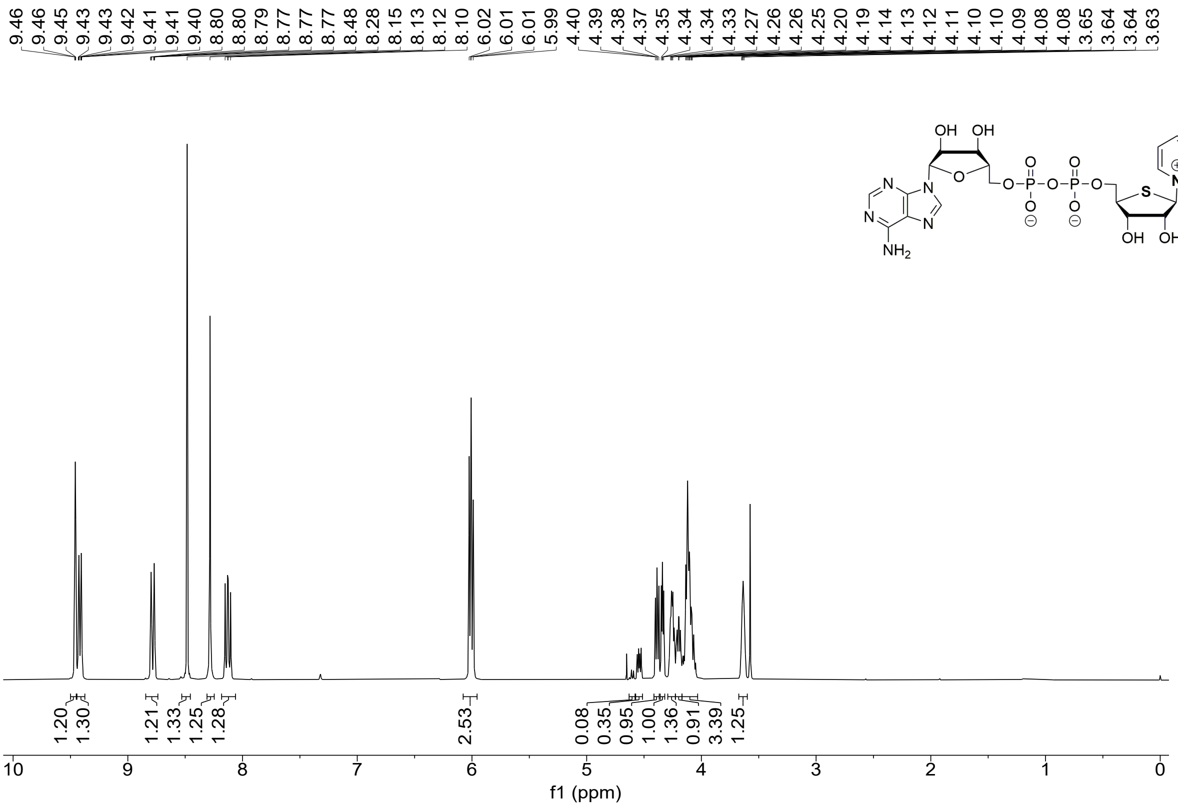

^{13}C-NMR (75 MHz, D_2O)

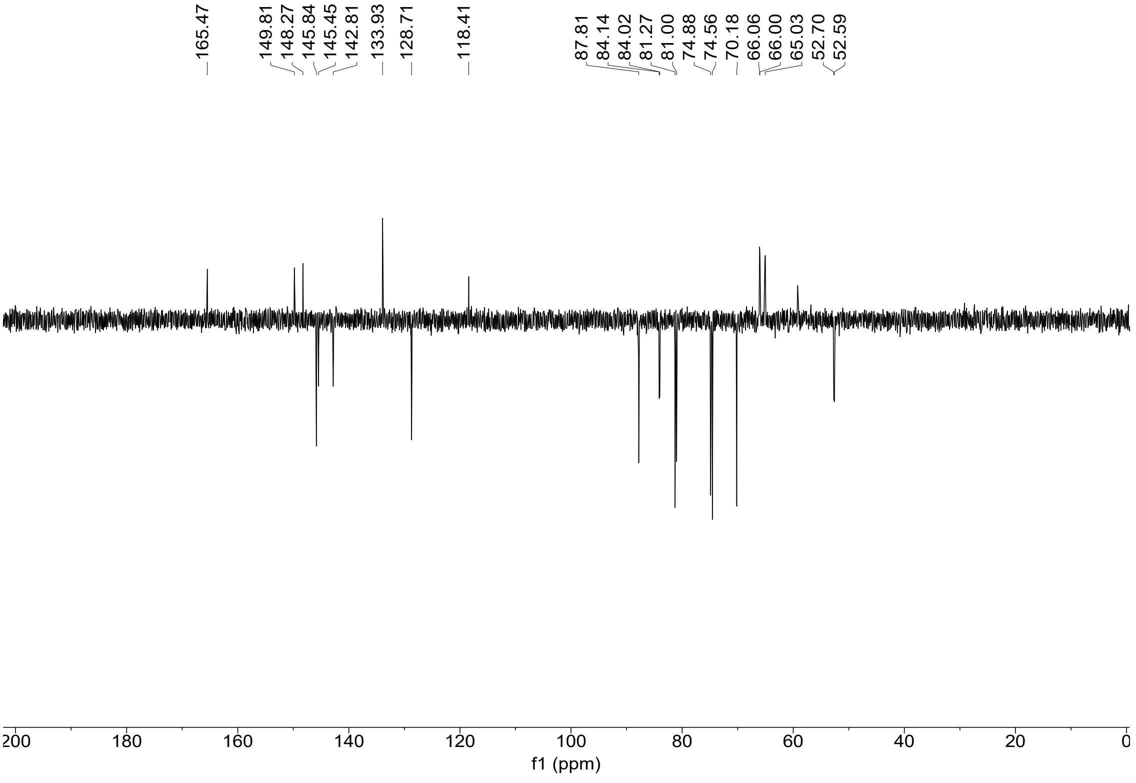

^{31}P-NMR (121 MHz, D_2O)

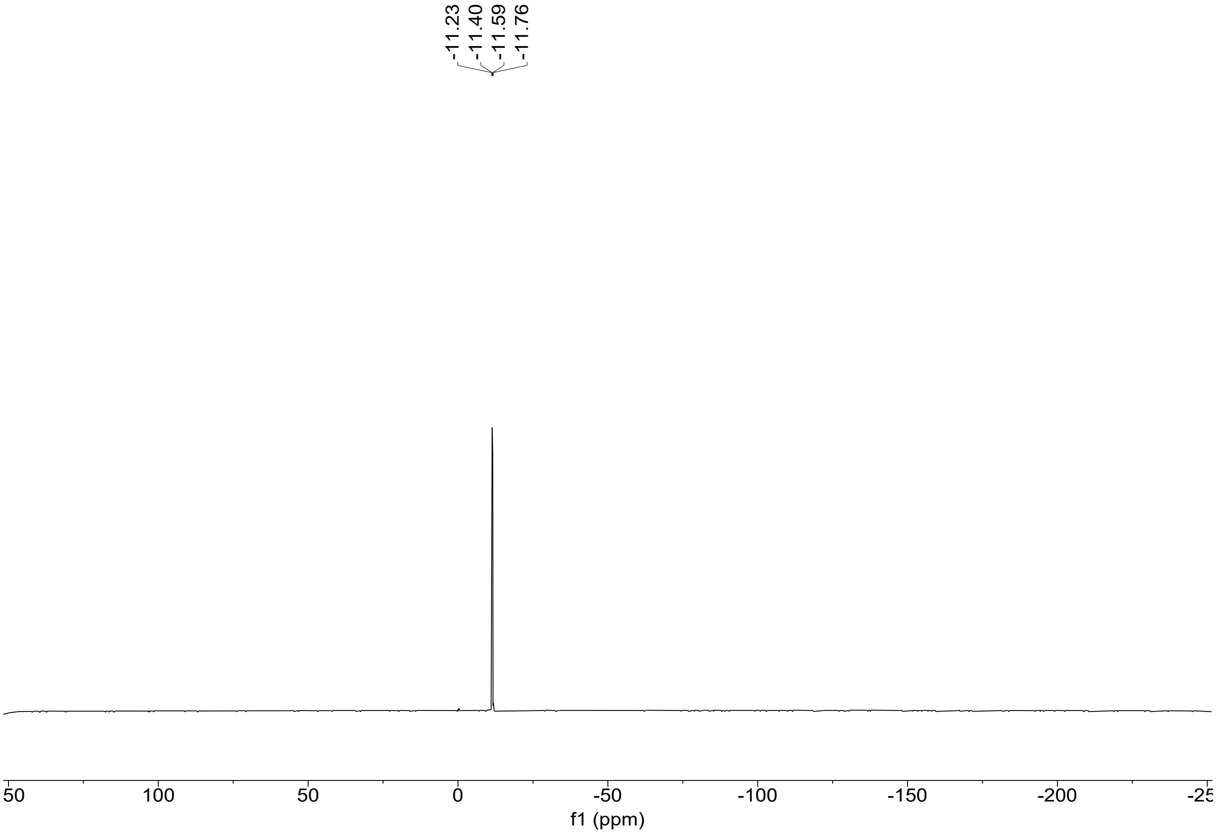

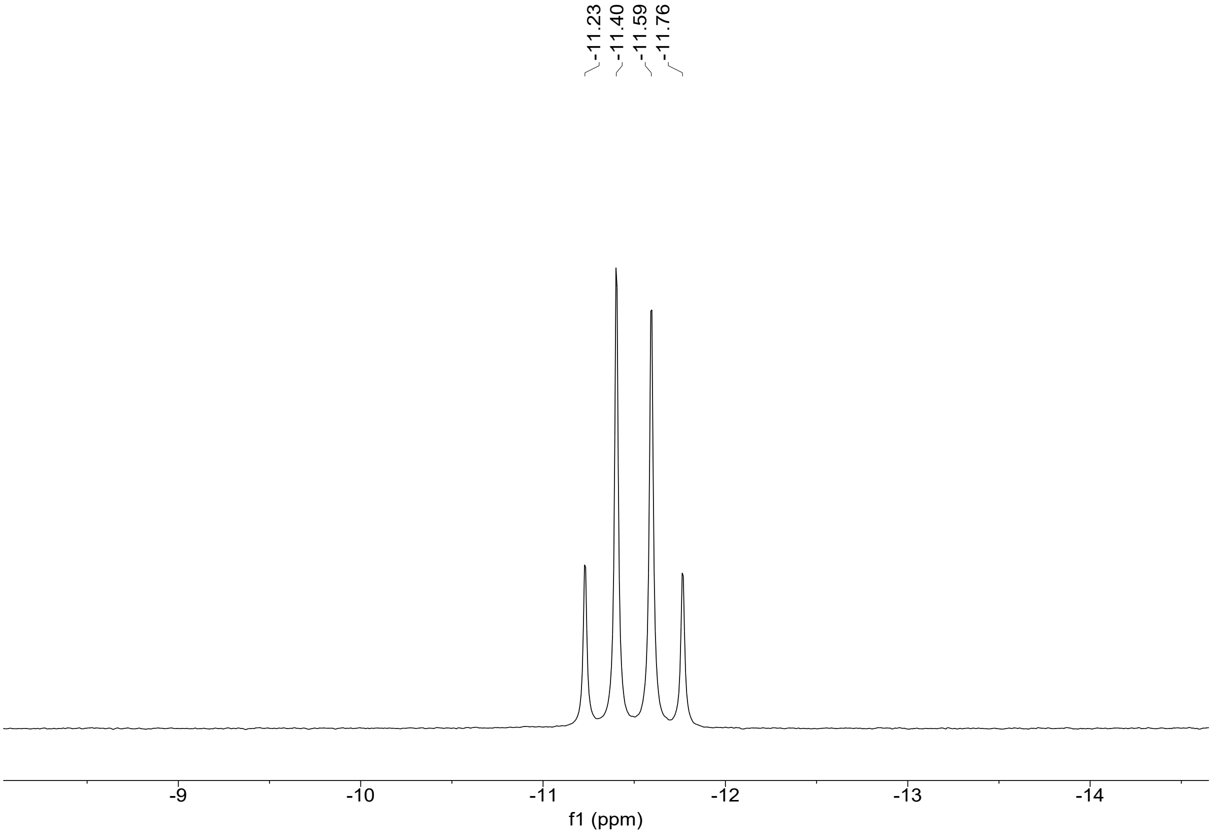

BAD (3)

^{1}H-NMR (300 MHz, D_2O)

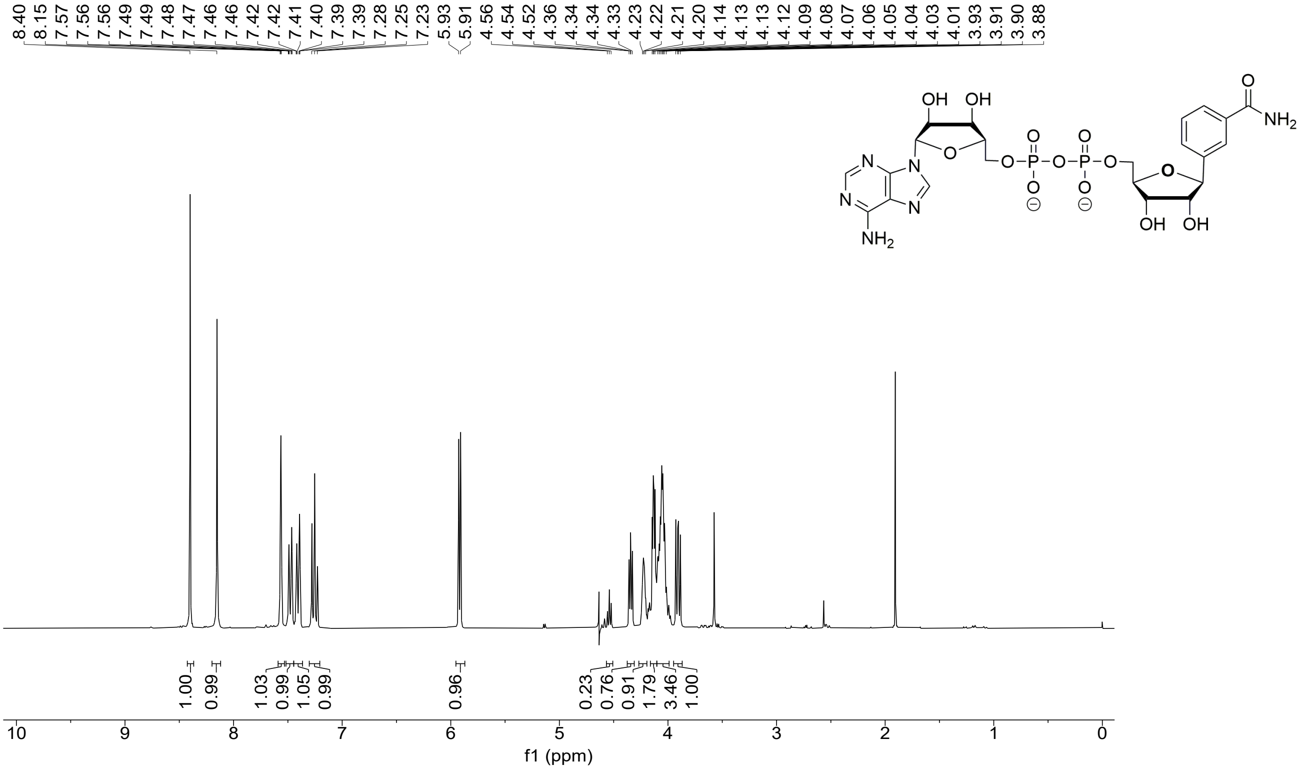

^{13}C-NMR (75 MHz, D_2O)

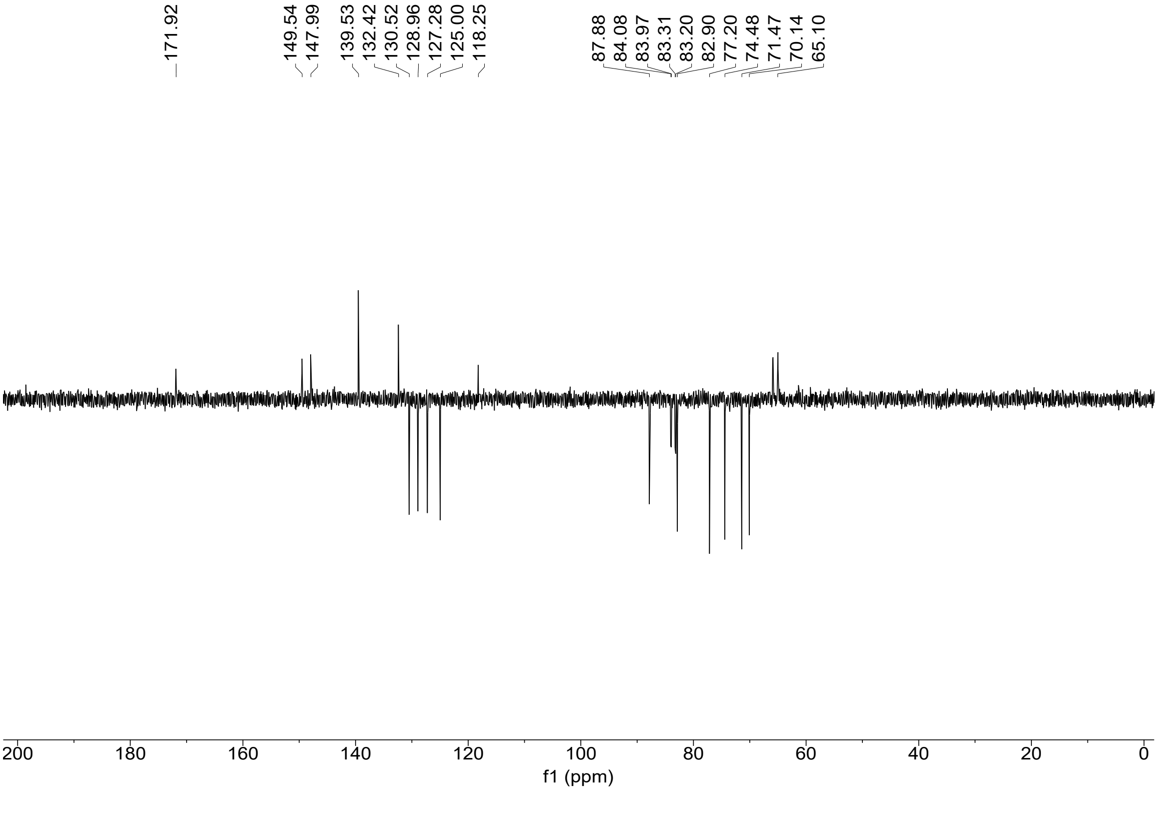

^{31}P-NMR (121 MHz, D_2O)

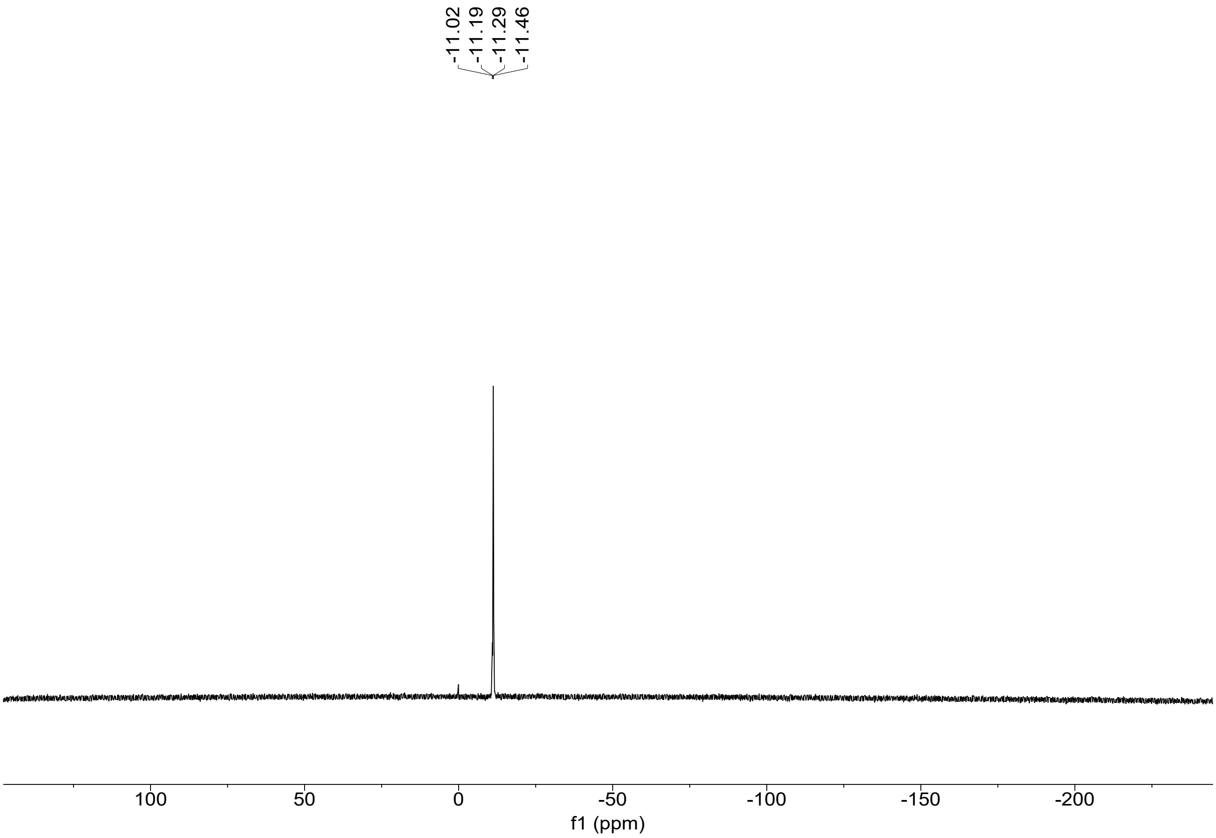

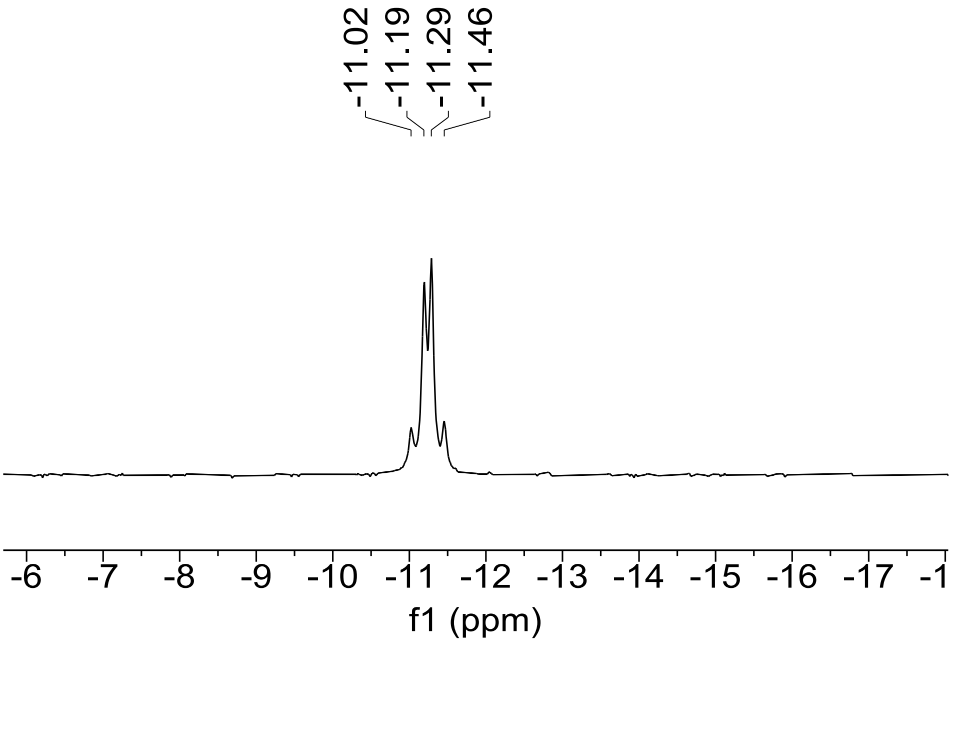

c-NR (4)

^{1}H-NMR (300 MHz, D_2O)

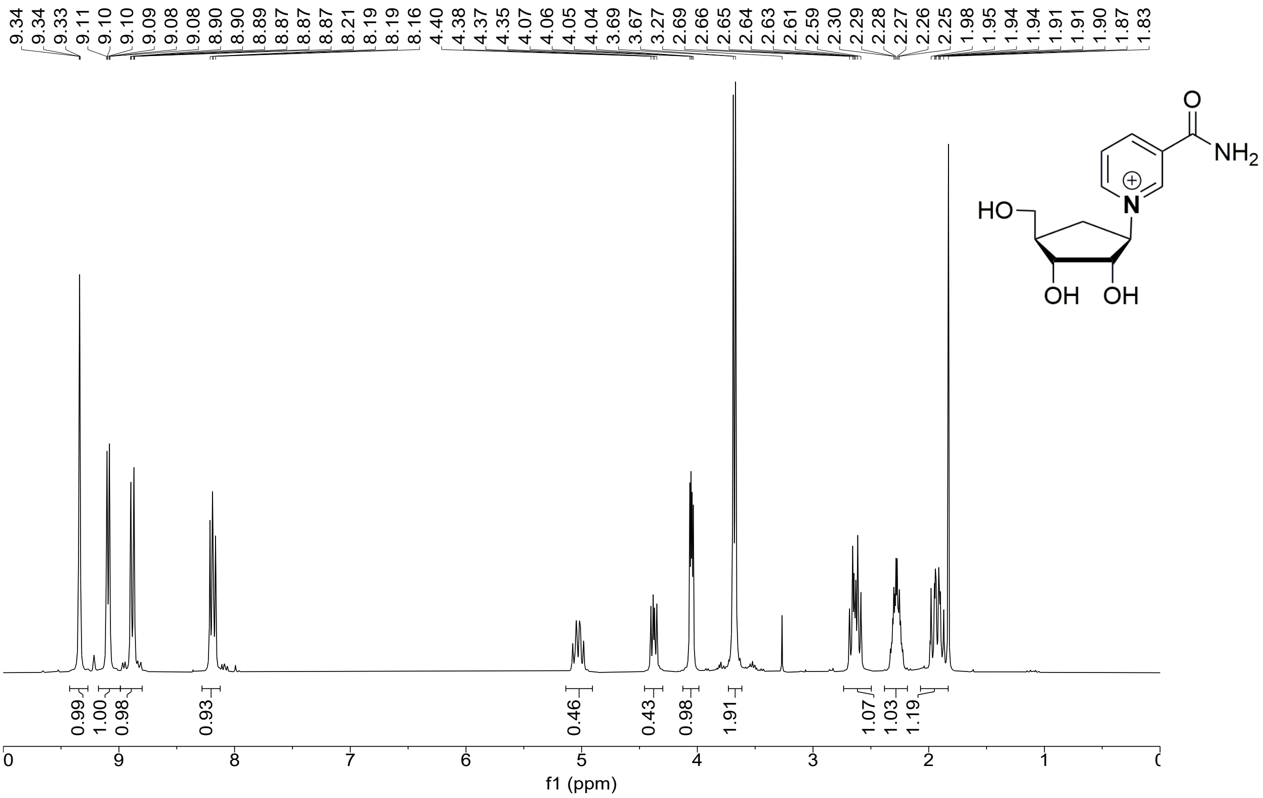

^{13}C-NMR (75 MHz, D_2O)

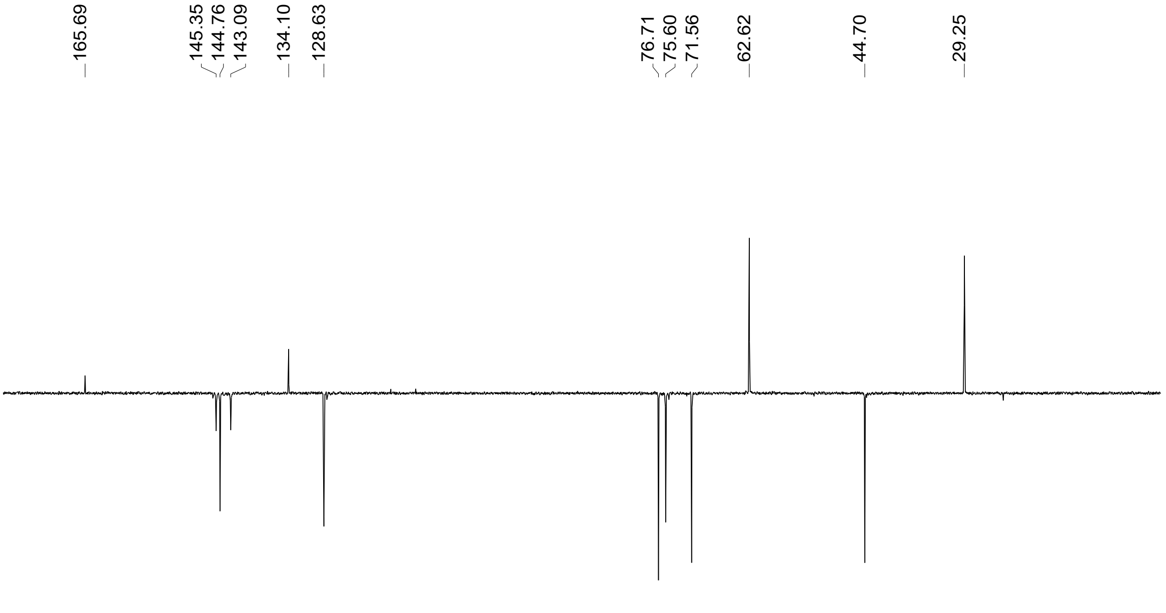

4-thio- α-D-nicotinamide ribose (5α)

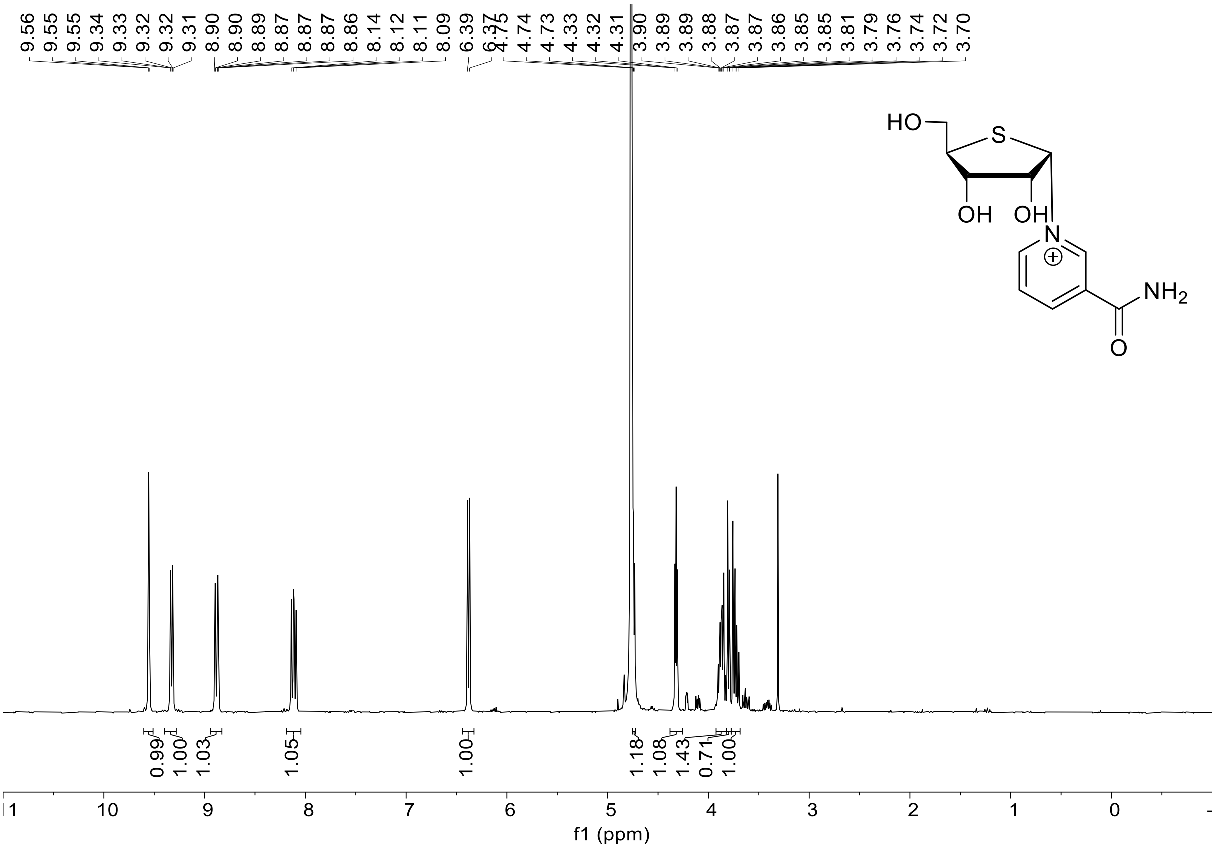

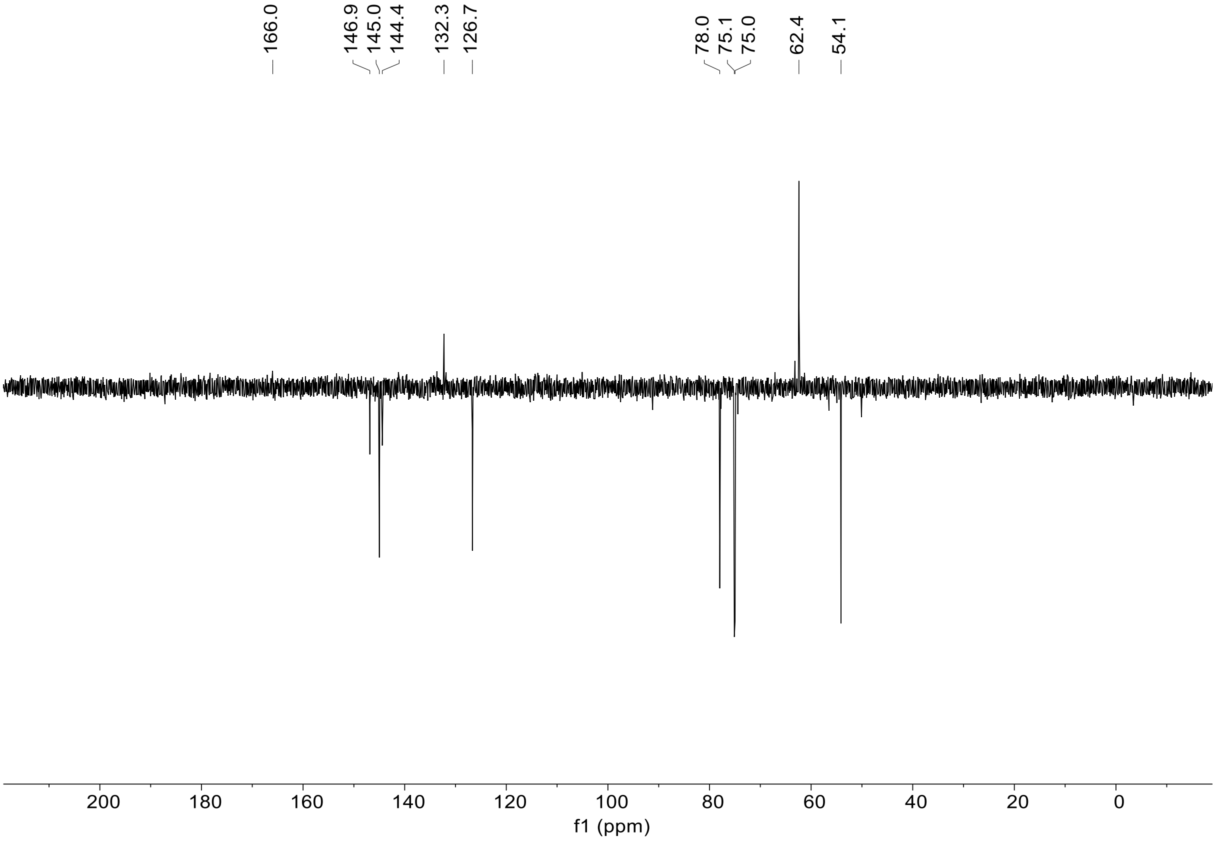

4-thio- β-D-nicotinamide ribose (5β)

^{1}H-NMR (300 MHz, D_2O)

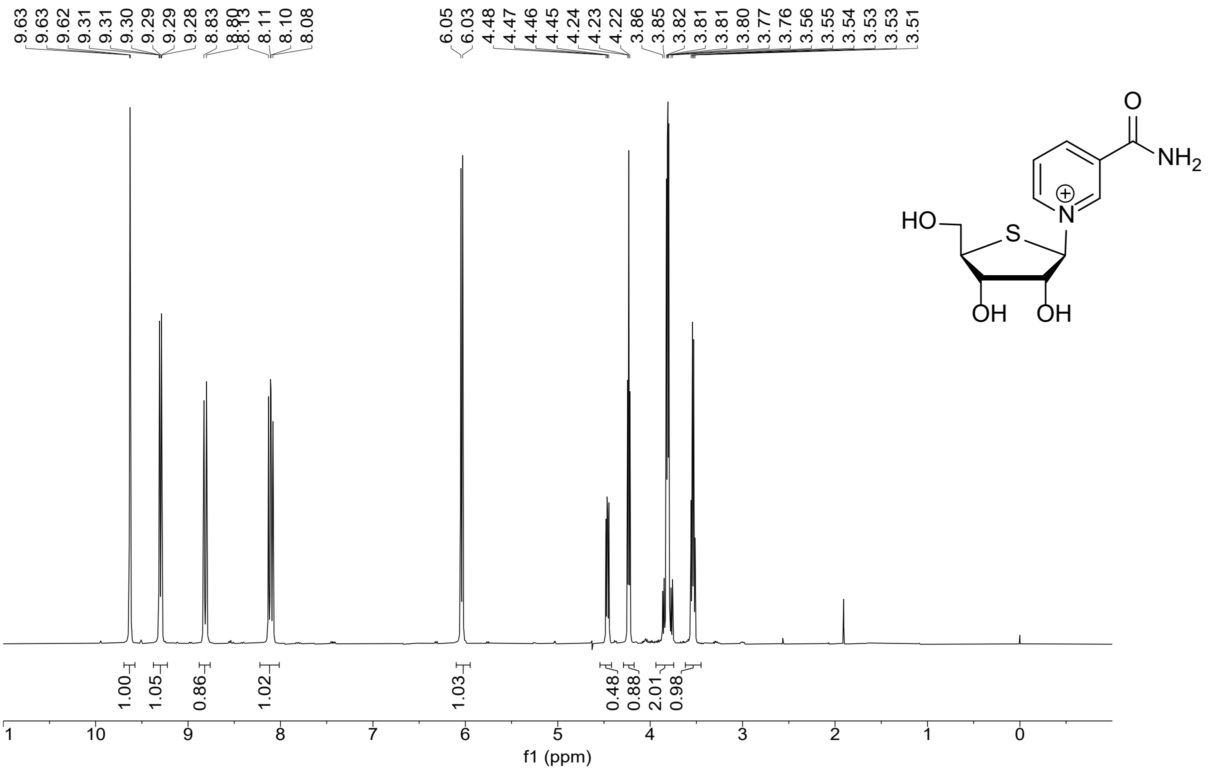

^{13}C-NMR (75 MHz, D_2O)

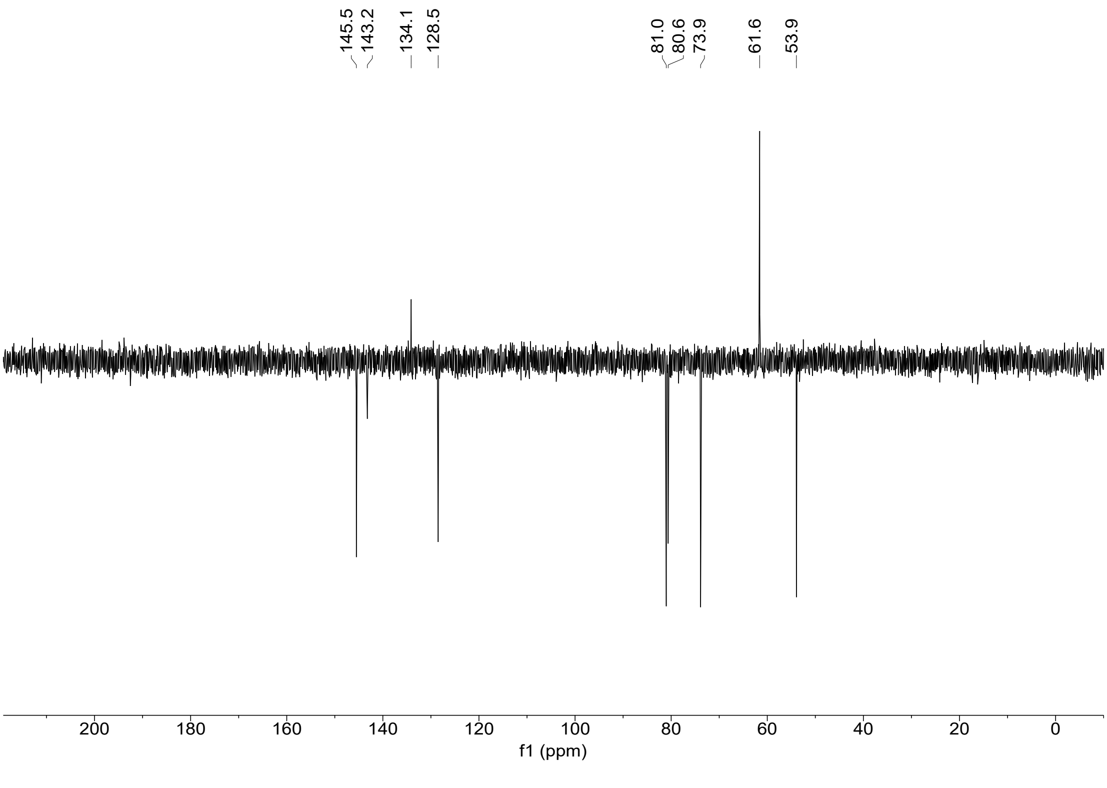

B-NR (6)

^{1}H-NMR (300 MHz, MeOD-*d4*)

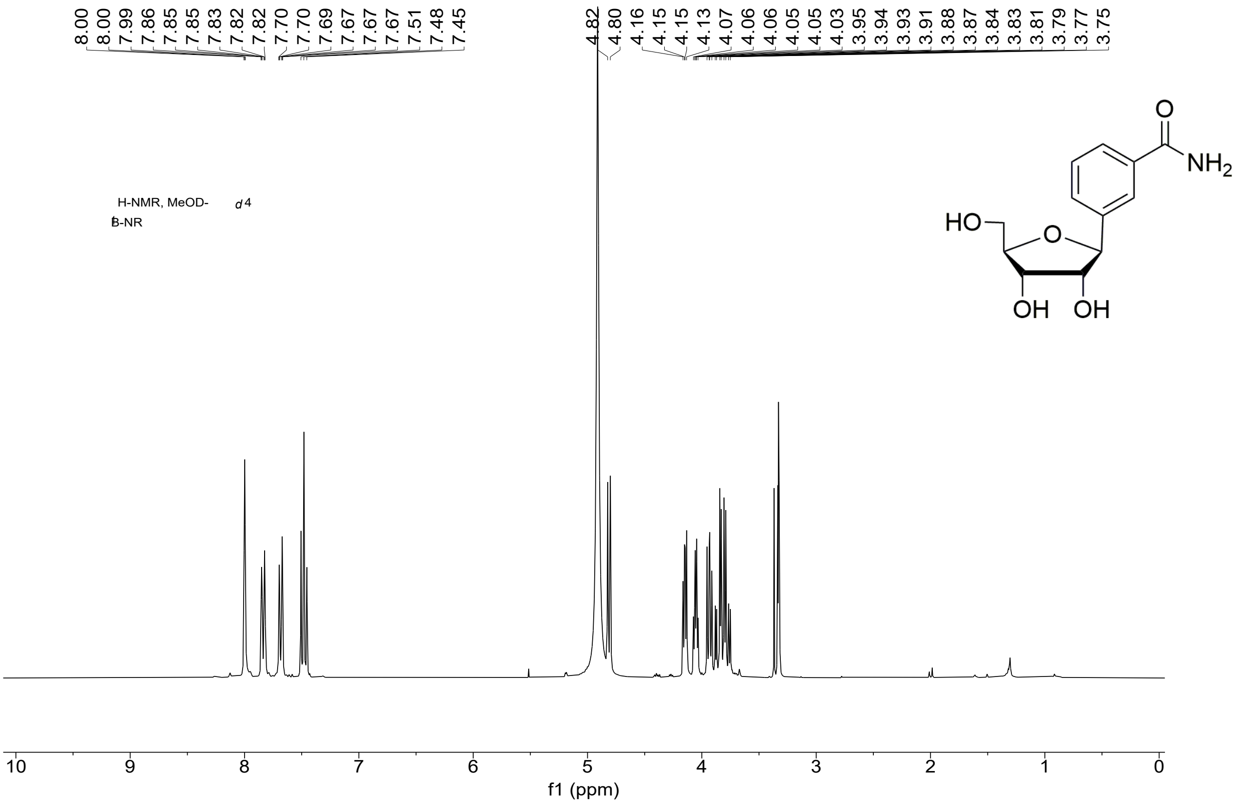

^{13}C-NMR (75 MHz, MeOD-*d4*)

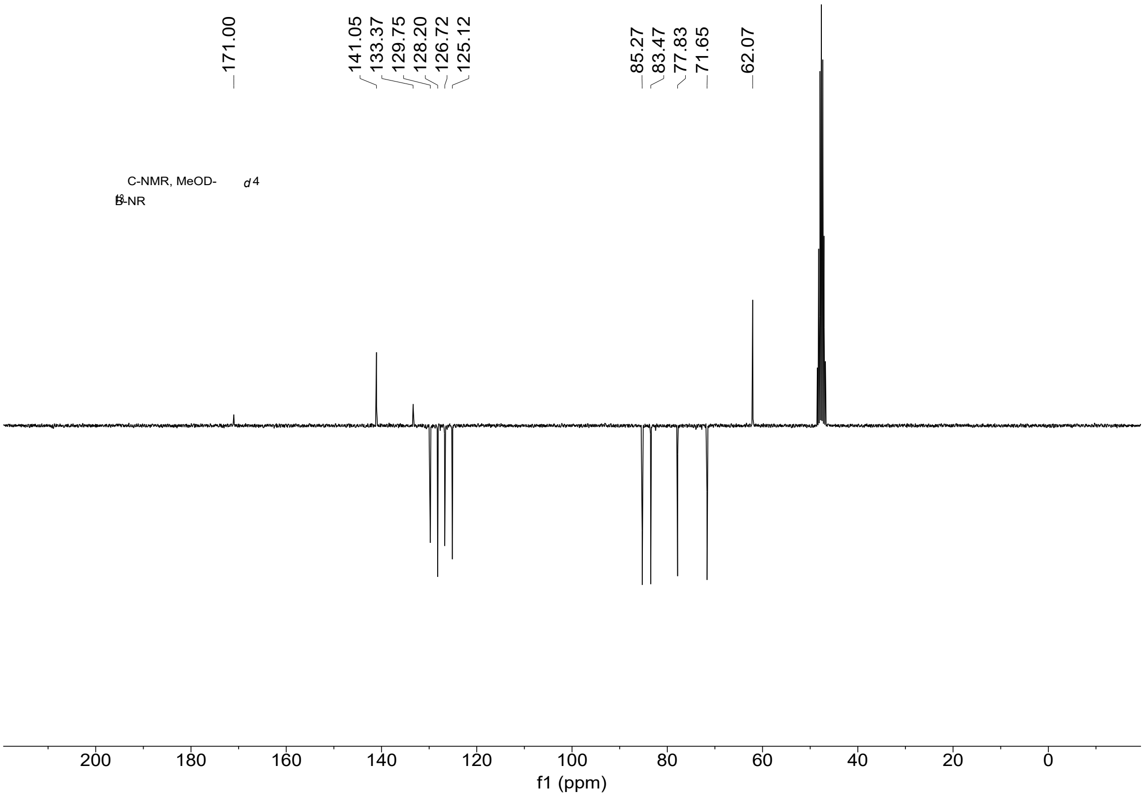

1,4-Anhydro-2,3,5-O-tri-paramethoxybenzyl-4-thio-D-ribitol (13)

^{1}H-NMR (400 MHz, $CDCl_3$)

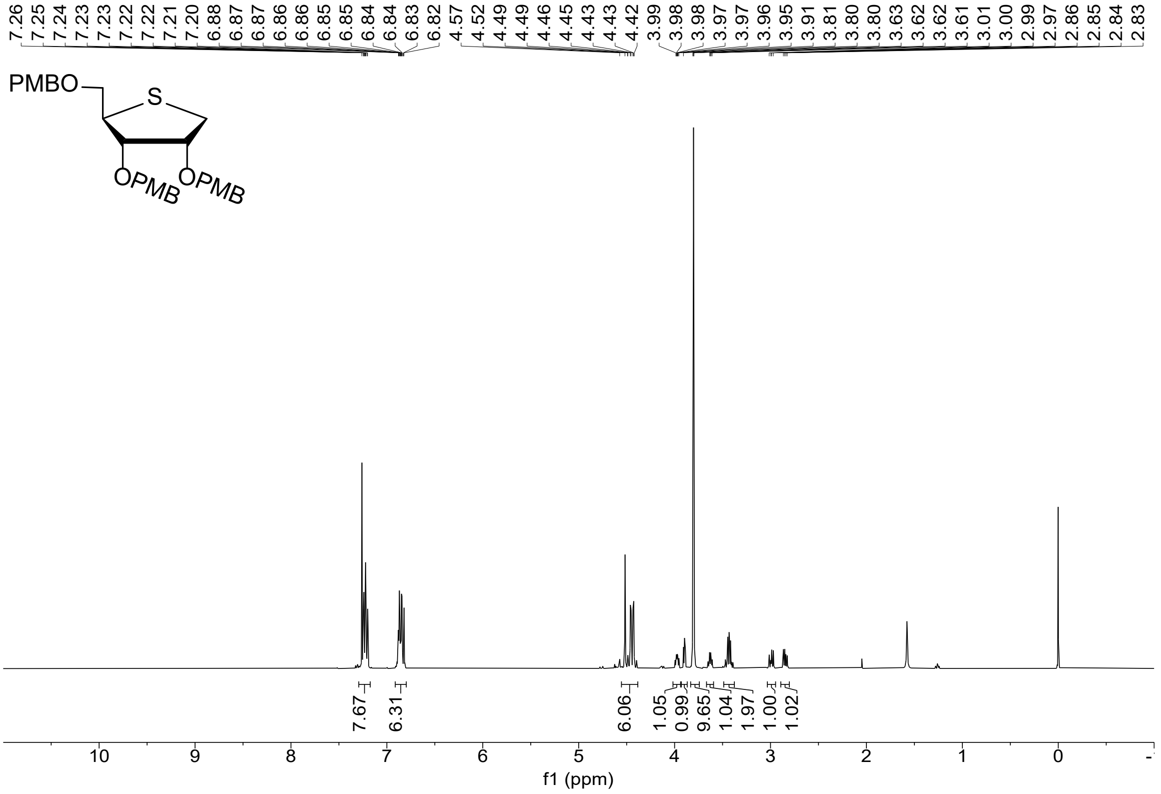

^{1}H-NMR (101 MHz, $CDCl_3$)

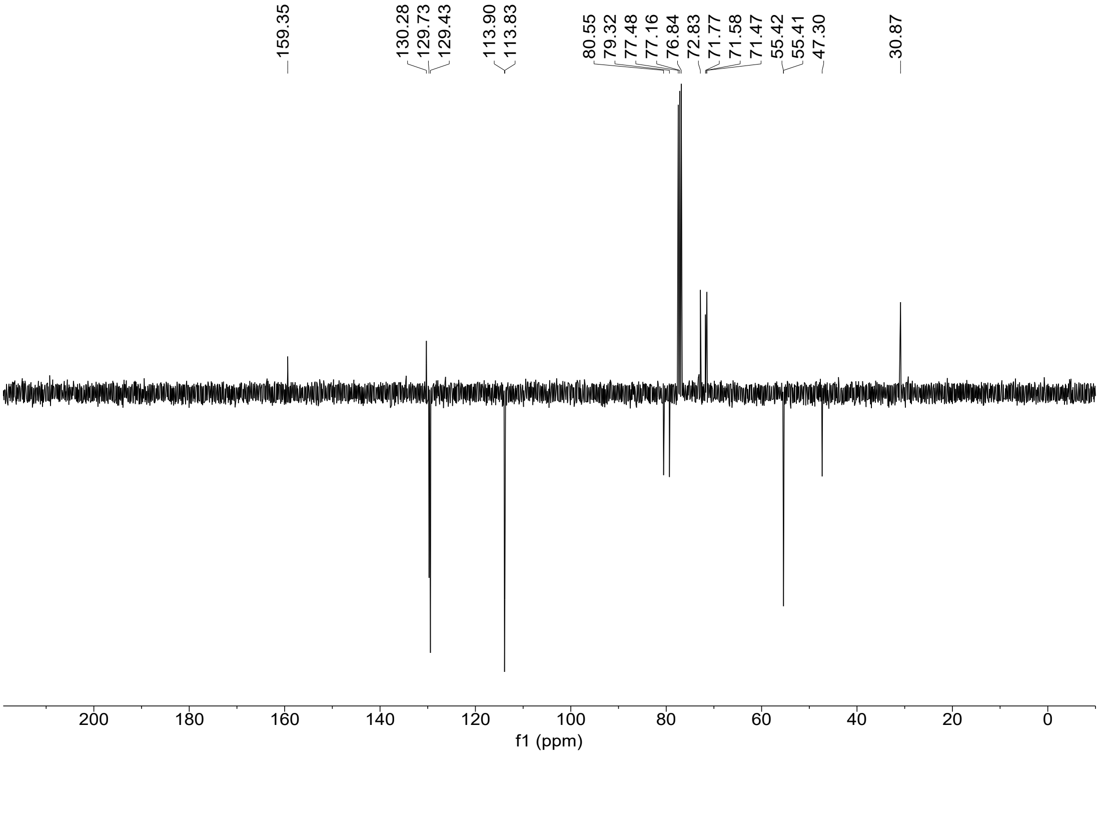

1,4-Anhydro-4-thio-D-ribitol (14)

^{1}H-NMR (400 MHz, MeOD-*d4*)

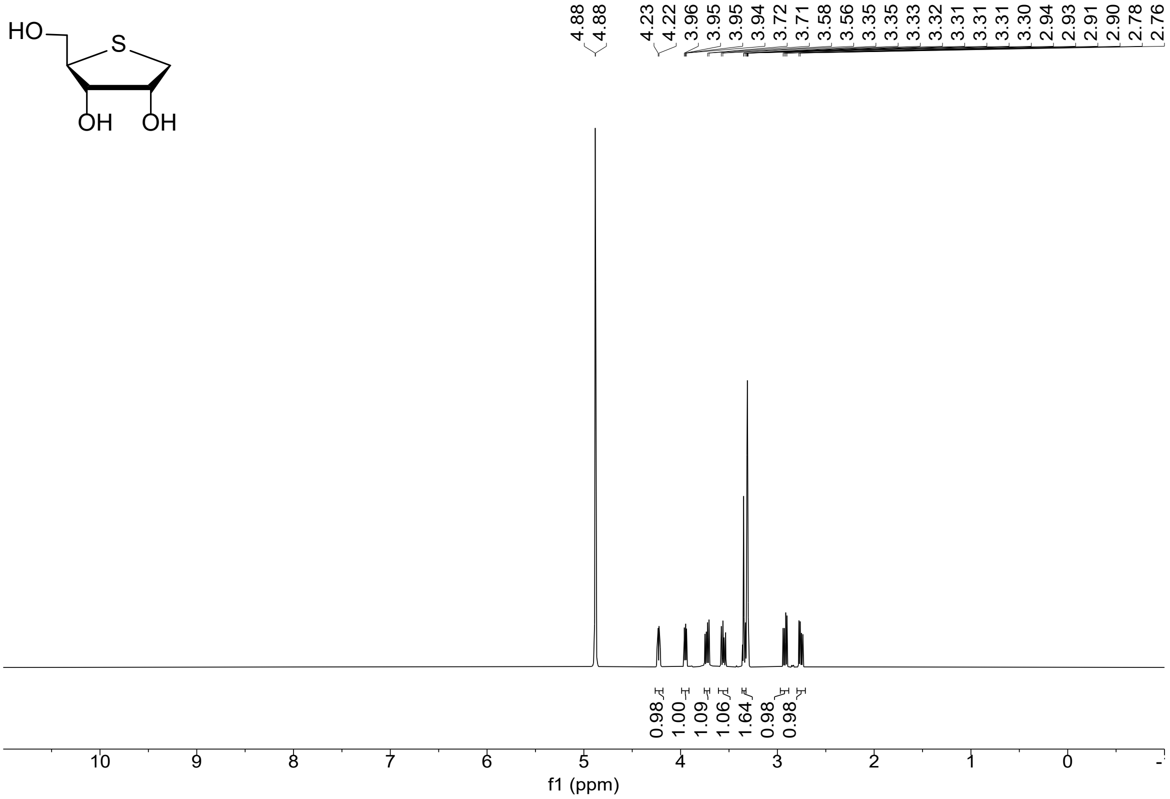

^{13}C-NMR (101 MHz, MeOD-*d4*)

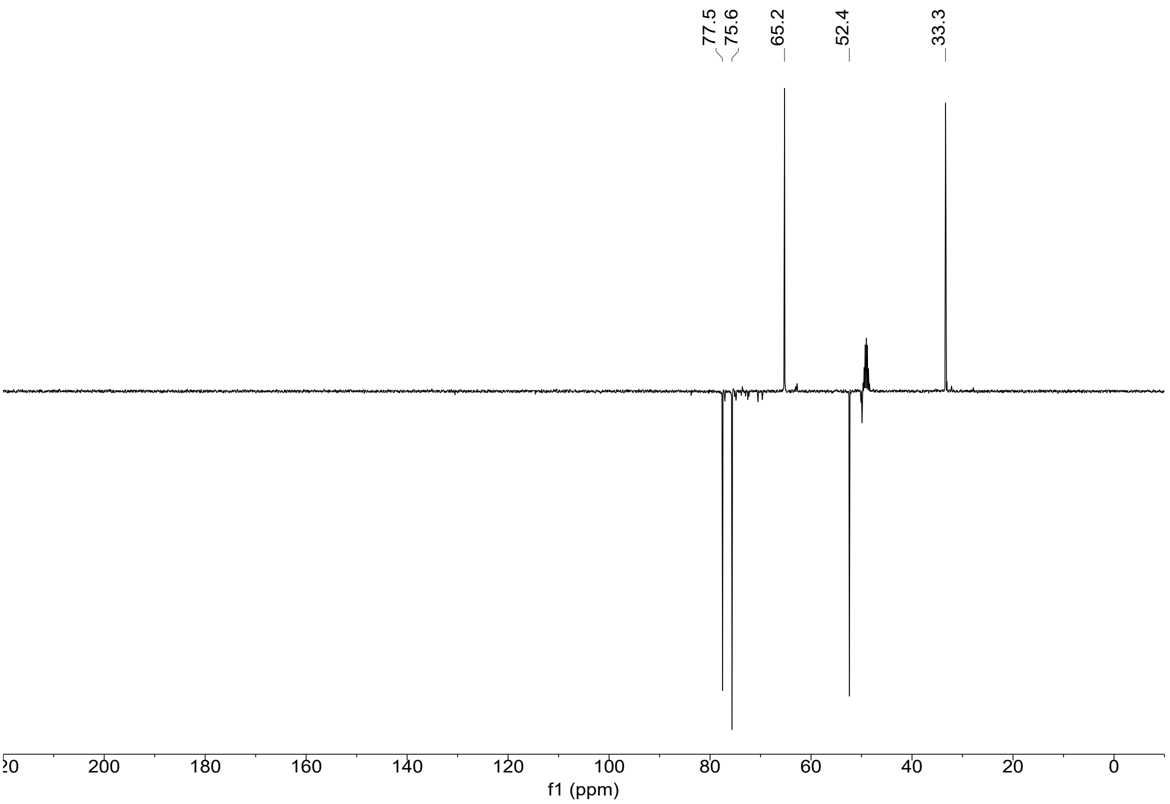

1,4-Anhydro-5-O-tert-butyldiphenylsilyl-4-thio-D-ribitol (15)

^{1}H-NMR (400 MHz, $CDCl_3$)

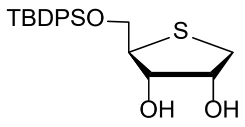

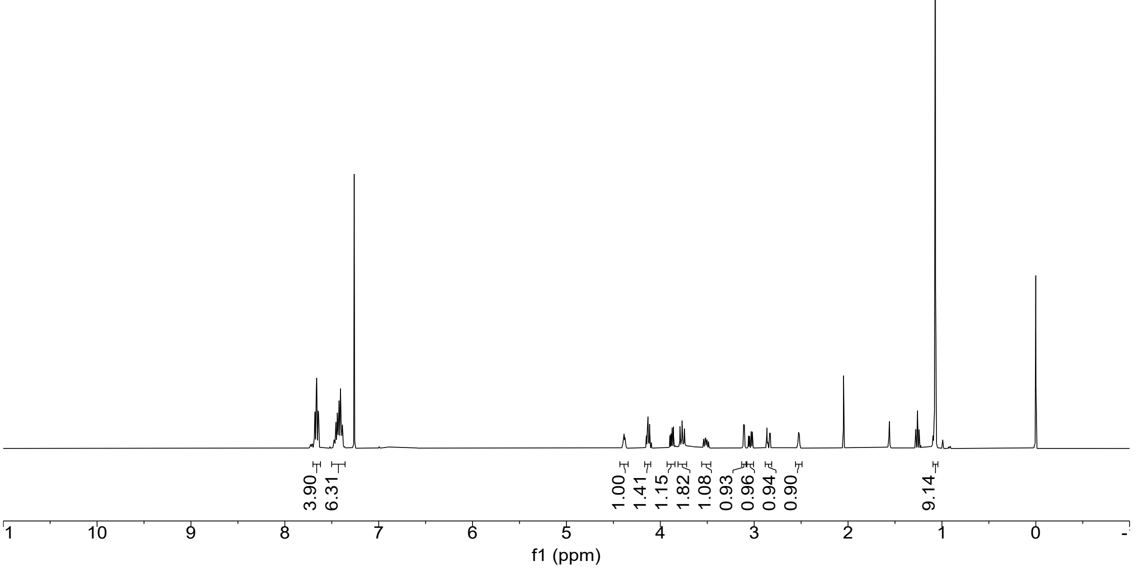

^{13}C-NMR (101 MHz, $CDCl_3$)

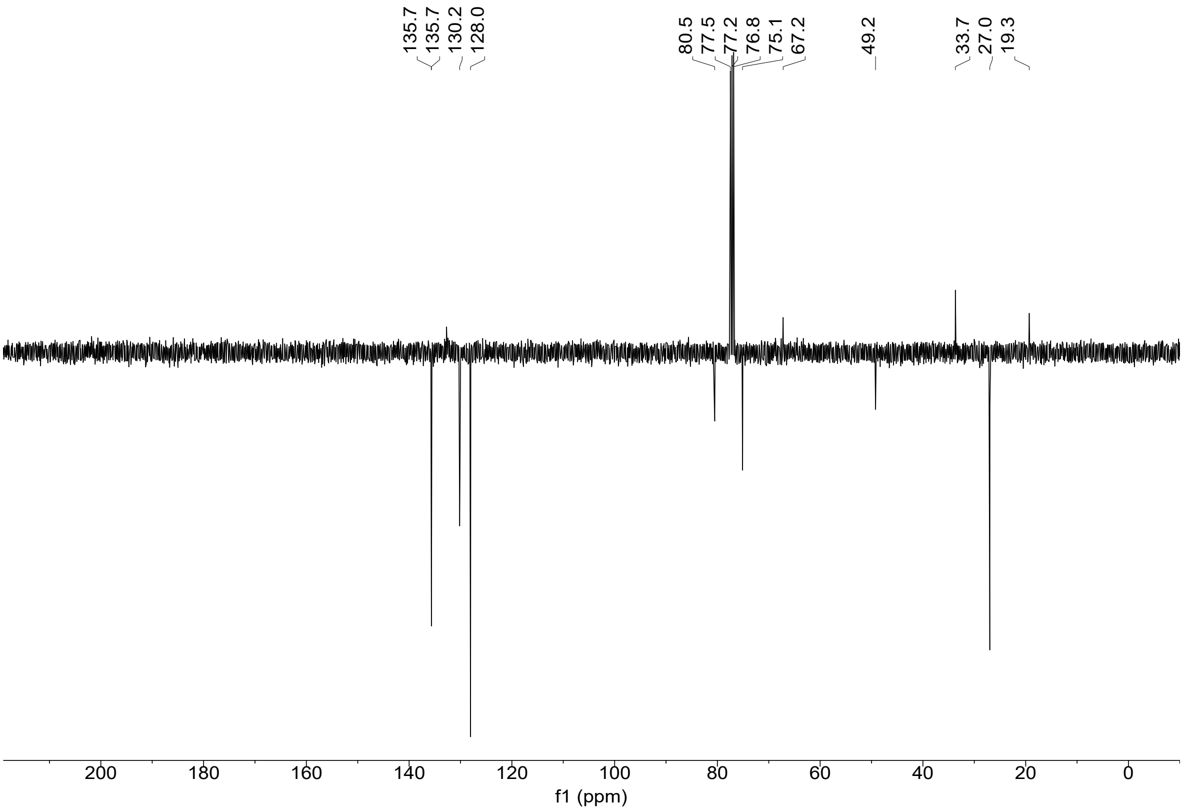

1,4-Anhydro-2,3-bis-O-acetyl-5-O-tert-butyldiphenylsilyl-4-thio-D-ribitol (16)

^{1}H-NMR (500 MHz, $CDCl_3$)

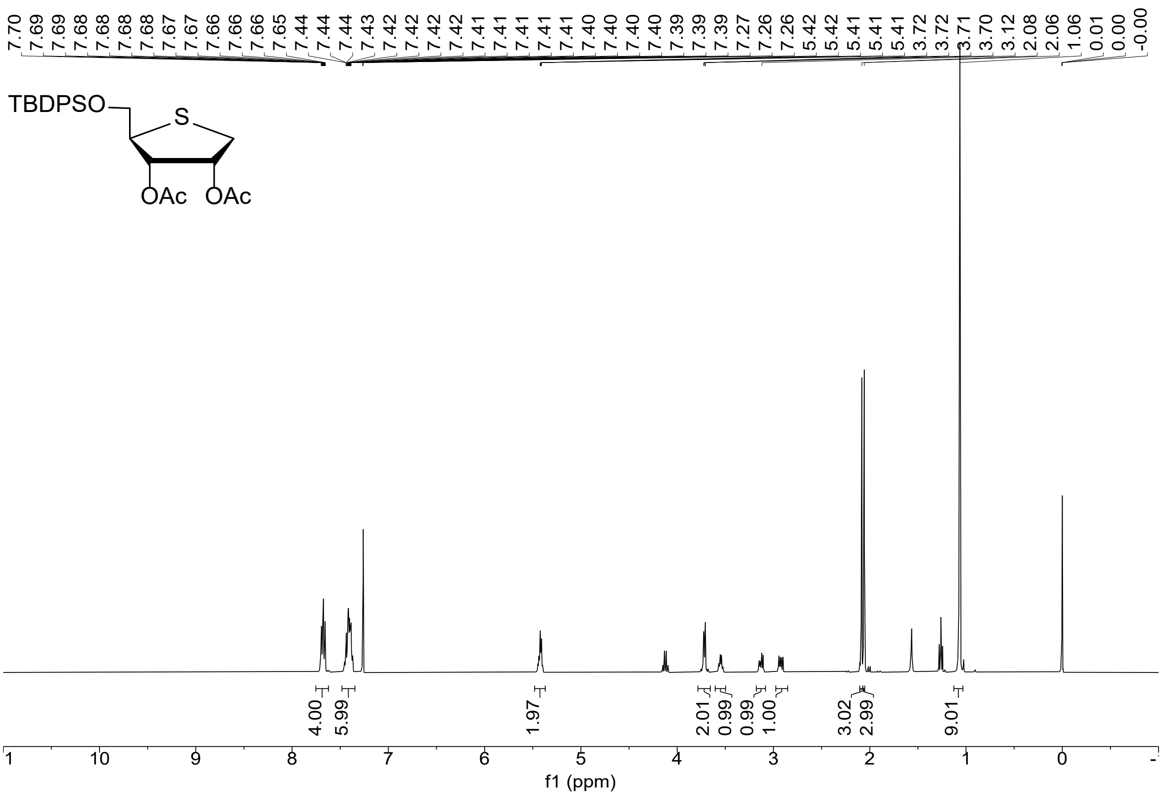

^{13}C-NMR (126 MHz, $CDCl_3$)

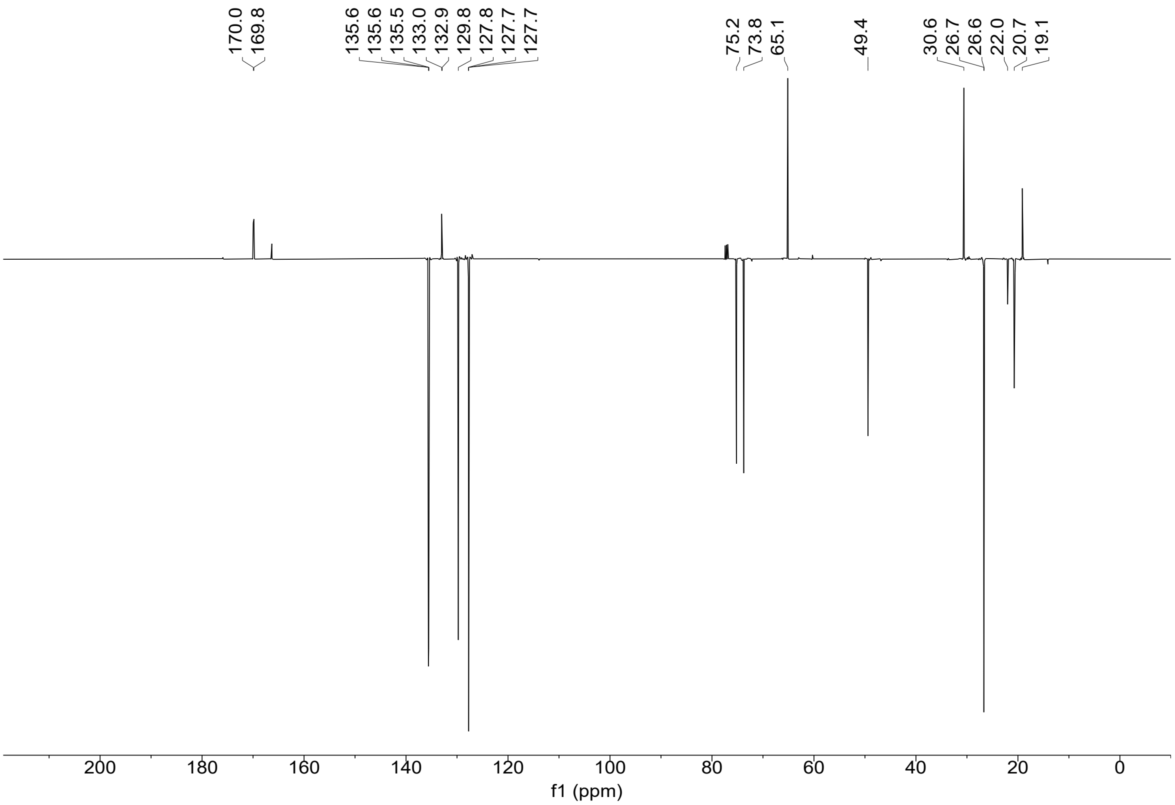

1,2,3-tri-O-Acetyl-5-O-tert-butyldiphenylsilyl-4-thio-α,β-D-ribofuranoside (18)

^{1}H-NMR (400 MHz, $CDCl_3$)

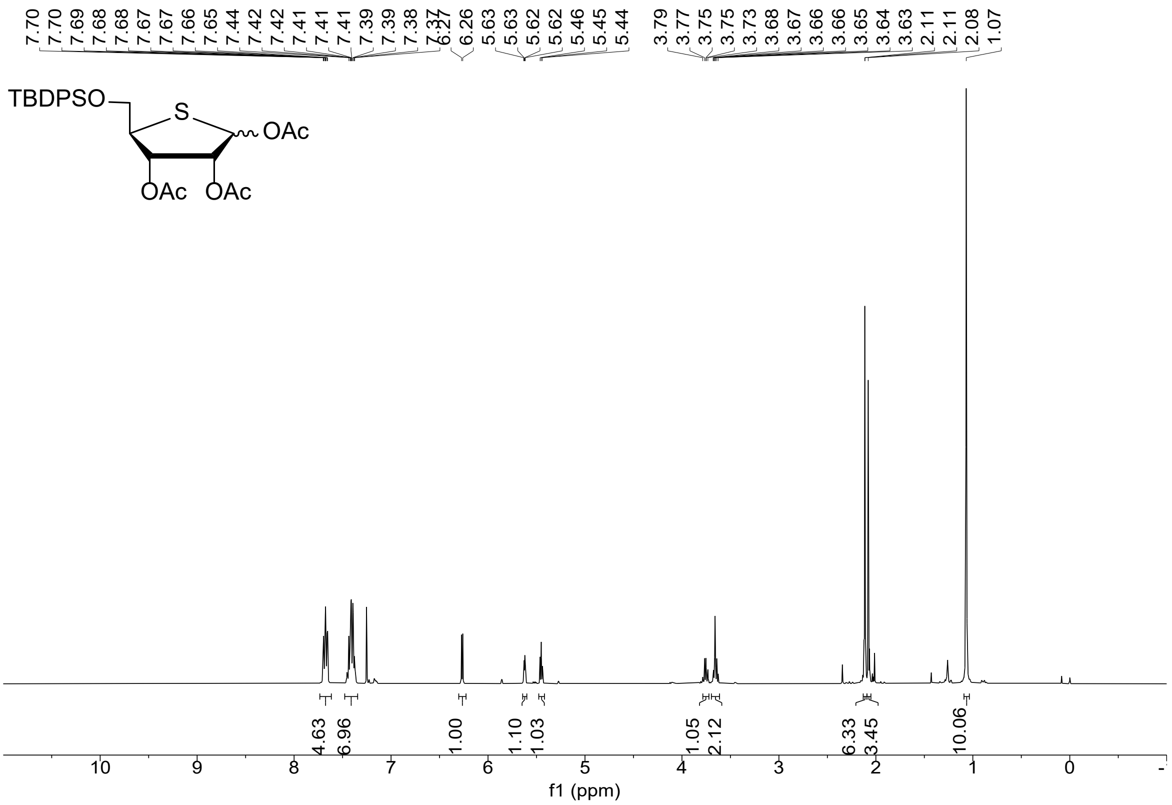

^{13}C-NMR (101 MHz, $CDCl_3$)

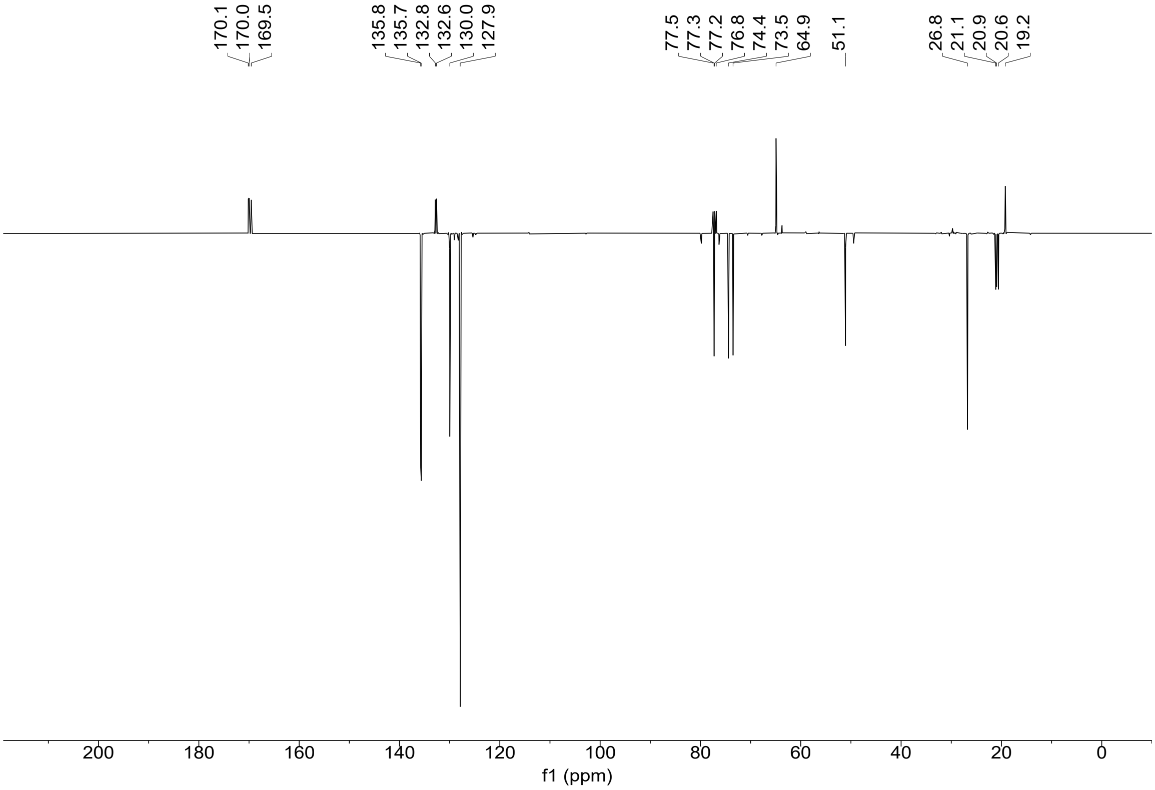

2,3-tri-O-acetyl-5-O-tert-butyldiphenylsilyl-4-thio- α, β-D-nicotinamide ribose (19)

^{1}H-NMR (400 MHz, MeOD-*d4*)

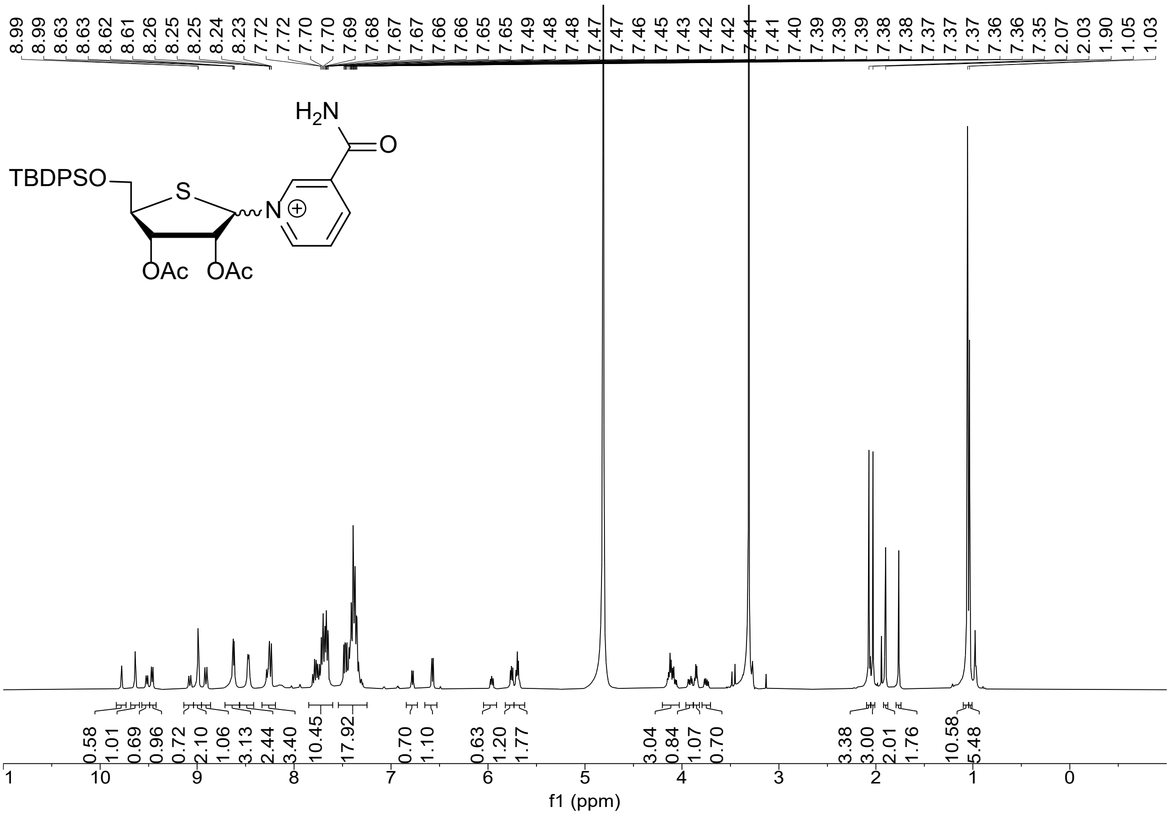

^{13}C-NMR (101 MHz, MeOD-*d4*)

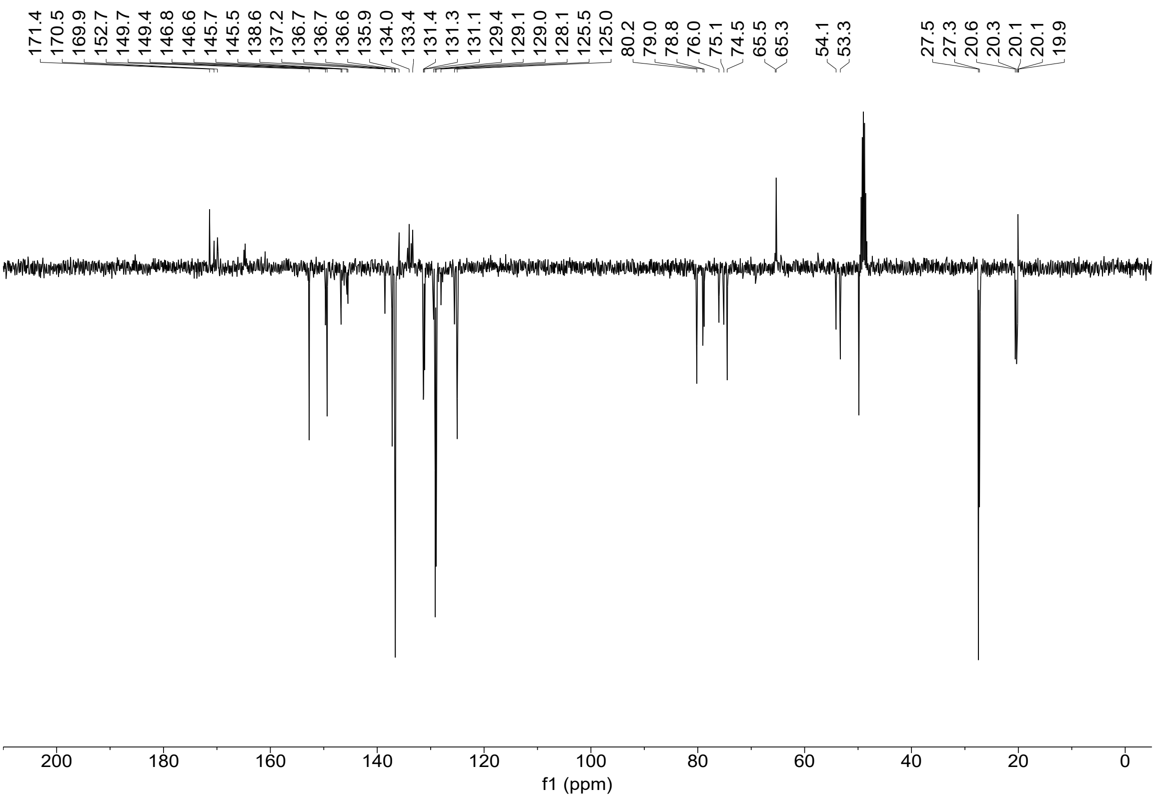

2,3-tri-O-acetyl -4-thio- α, β-D-nicotinamide ribose (20)

^{1}H-NMR (600 MHz, MeOD-*d4*)

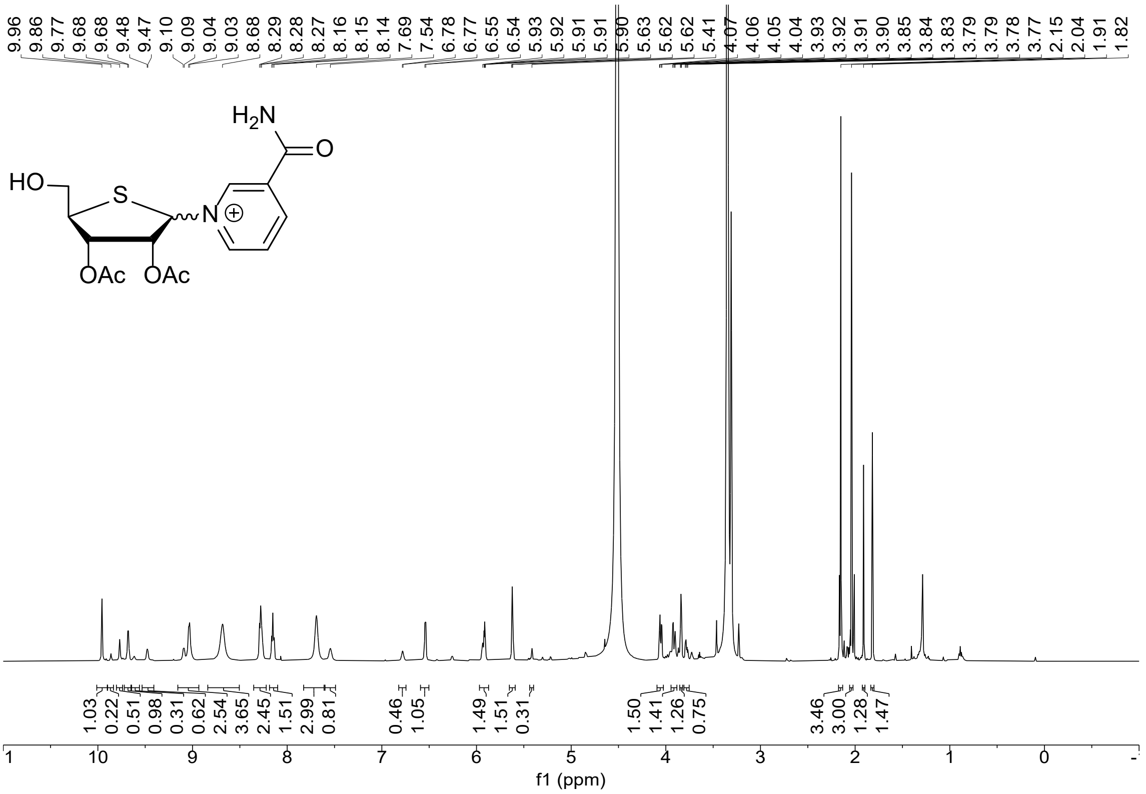

^{13}C-NMR (150 MHz, MeOD-*d4*)

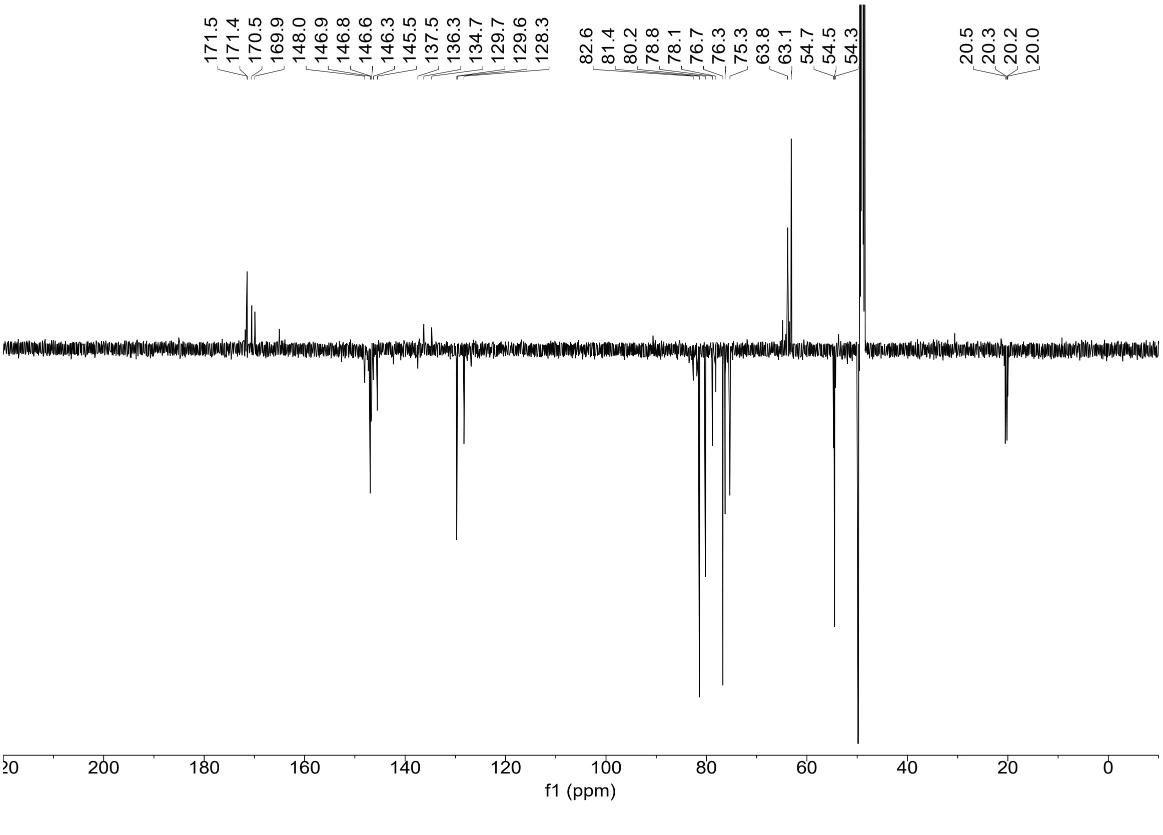

Supplement: Supplementary file 1 — Supplementary [file CBIC-21-2903-s001.pdf]
